# Longitudinal changes and risk factors of *Opisthorchis viverrini* infection after selective praziquantel treatment: evidence from urine antigen assay and fecal examination in an endemic community in Northeast Thailand

**DOI:** 10.1371/journal.pone.0352854

**Published:** 2026-07-06

**Authors:** Kulthida Y. Kopolrat, Parichart Boueroy, Ratanee Kammoolkon, Opal Pitaksakulrat, Patiwat Yasaka, Chanika Worasith, Phattharaphon Wongphutorn, Chatanun Eamudomkarn, Chompunoot Wangboon, Nattaya Watwiengkam, Watcharin Promkhwan, Paiboon Sithithaworn

**Affiliations:** 1 Faculty of Public Health, Kasetsart University Chalermphrakiat Sakon Nakhon Province Campus, Sakon Nakhon, Thailand; 2 Cholangiocarcinoma Research Institute, Khon Kaen University, Khon Kaen, Thailand; 3 Department of Parasitology, Faculty of Medicine, Khon Kaen University, Khon Kaen, Thailand; 4 Faculty of Management Technology, Rajamangala University of Technology Isan, Surin Campus, Surin, Thailand; 5 Department of Adult Nursing, Faculty of Nursing, Khon Kaen University, Khon Kaen, Thailand; 6 Regional Medical Sciences Center 4 Saraburi, Department of Medical Sciences, Ministry of Public Health, Thailand; 7 School of Preclinical Sciences, Institute of Science, Suranaree University of Technology, Nakhon Ratchasima, Thailand; 8 Faculty of Veterinary Sciences, Mahasarakham University, MahaSarakham, Thailand; 9 Nhonghin Subdistrict Health Promoting Hospital, Kalasin, Thailand; Charles University: Univerzita Karlova, CZECHIA

## Abstract

*Opisthorchis viverrini* infection remains an important public health problem in Southeast Asia, particularly among rural populations in Northeast Thailand. Despite control efforts, transmission remains uninterrupted due to persistent behavioral and environmental factors. To better understand the longitudinal patterns of opisthorchiasis after selective praziquantel treatment, this study aimed to determine the incidence and reinfection rates in the study population and identify risk factors associated with *O. viverrini* infection. Based on a prospective study, the status of opisthorchiasis was monitored in a cohort of participants (n = 612) in Northeast Thailand using the formalin-ethyl acetate concentration technique (FECT) and *O. viverrini* antigen detection in urine by Enzyme-linked immunosorbent assay (ELISA). The baseline prevalence of *O. viverrini* infection was 41.0% by urine antigen assay, compared to 8.1% by FECT. Over the 24-week study period, the calculated incidence of infection was 64.6/100 person-years, and the reinfection rate after PZQ treatment, as measured by urine ELISA, was 63.7/100 person-years. Based on FECT, a tenfold lower incidence (7.5/100 person-years) and reinfection rate (5.9/100 person-years) were observed. Risk factor analysis identified raw fish consumption in the previous 6 months (aRR = 7.52; p < 0.001), frequent raw fish consumption (>10 times per month) (aRR = 3.60; p < 0.001), and previous praziquantel treatment aRR = 1.49; p = 0.032) as significant risk factors for infection. The results demonstrated that the persistence of opisthorchiasis prevalence is driven mainly by the incidence of infection and reinfection after chemotherapy, as assessed by urine antigen assay and FECT. Behavioral risk factors, particularly the consumption of raw fish, remain the primary risk of parasite transmission. Therefore, comprehensive intervention measures consisting of sensitive diagnostics, drug treatment, and culturally appropriate interventions are required.

## Introduction

Opisthorchiasis is a neglected tropical disease caused by an infection with the foodborne liver fluke, *Opisthorchis viverrini*. It remains a significant public health issue in many parts of Southeast Asia, particularly in Thailand, the Lao People’s Democratic Republic (Lao PDR), Cambodia, and Vietnam [1–3]. *O. viverrini* infection prevalence is up to 70% in some areas, with estimates of up to 10 million human infections in the lower Mekong region [4,5]. Based on its strong association with cholangiocarcinoma (CCA), *O. viverrini* has been classified as a Group I biological carcinogen in humans by the International Agency for Research on Cancer [6]. The clinical consequences of chronic opisthorchiasis include chronic inflammation and hepatobiliary pathology, particularly intrahepatic CCA [7,8]. As *O. viverrini* infection plays a fundamental role in the induction of CCA and resulting fatalities, a comprehensive strategy for the prevention, control, and elimination of *O. viverrini* is a prerequisite for reducing the incidence of CCA [9].

A prospective risk factor for *O. viverrini* infection is the consumption of raw or undercooked freshwater fish containing infective metacercariae [10,11]. The transmission cycle is perpetuated by parasite eggs in human or animal feces that contaminate freshwater sources, including those containing *Bithynia* snails and freshwater fish [3]. This dietary behavior is deeply embedded in the food culture of northeast Thailand and, more generally, the lower Mekong region [10,12]. The prevalence is high in northern and northeastern Thailand, associated with widespread infection in fish intermediate hosts [13,14]. Despite prospective control efforts, the prevalence persisted due to reinfection after chemotherapeutic control [15–19]. Widespread drug treatment may also lead to complacency, resulting in continued consumption of raw fish to perpetuate *O. viverrini* transmission [20]. Particularly, repeated exposure to infection has been hypothesized to increase the risk of CCA development [11,21–24]. In addition to reinfection, the incidence of infection is another transmission parameter that has contributed to the resurgence of opisthorchiasis [15]. Currently, reports on the transmission dynamics of *O. viverrini,* incorporating both incidence and reinfection rates and associated risk factors in longitudinal studies, are not well documented [25]. This information is essential in understanding the current situation in transmission dynamics and is crucial for the development of an effective control strategy in opisthorchiasis

This study aimed to investigate longitudinal changes in *O. viverrini* infection following selective praziquantel treatment by assessing infection intensity, reinfection, and incidence, as well as identifying associated risk factors, using a urine antigen assay and fecal examination in an opisthorchiasis-endemic community in Northeast Thailand. The results from this study highlighted the major roles of reinfection and incidence of infection in shaping the transmission dynamics of opisthorchiasis. The use of the urine antigen assay offered advantages over the fecal examination in terms of sensitivity and the ease of sample handling and analysis. Further application in wider areas to visualize a more comprehensive transmission pattern to improve public health intervention measures is warranted.

## Materials and methods

### Ethics statement

The human experimental protocol was approved by the Kasetsart University Ethics Committee (reference number KUREC-CSC66/054). Only participants who provided written informed consent were included in the study. Participants who were diagnosed as *O. viverrini*-positive by FECT and/or urine antigen detection survey received a single oral dose of PZQ (40 mg/kg body weight). Participants who were found to be positive for other parasitic infections were given appropriate anthelmintic drugs.

### Study design and sample population

A prospective cohort study was conducted from April to November 2024 in an opisthorchiasis-endemic area in Khok Kruea sub-district, Nong Kung Si district, Kalasin, Northeastern Thailand. Participants were recruited from an endemic community using a simple random household sampling approach. Within the sampled households, the registry members were recruited as participants based on the inclusion criterion: (1) Male and female residents aged 15 years or older, (2) residing in the study area during the project operation, and (3) being in good general health. Written informed consent was obtained from all participants aged 18 years or older. For participants aged 15–17 years, written informed consent was obtained from their parents or legal guardians prior to participation in the study. After obtaining written informed consent, the project participants were interviewed using a structured questionnaire to collect demographic and lifestyle information, including socio-demographic characteristics (age, gender, level of education, and primary occupation), history of *O. viverrini* infection, history of previous praziquantel treatment, and lifestyle details. Moreover, participants’ raw fish consumption and sharing habits, as well as their fish procurement sites, were surveyed using a questionnaire.

**Fig 1:**
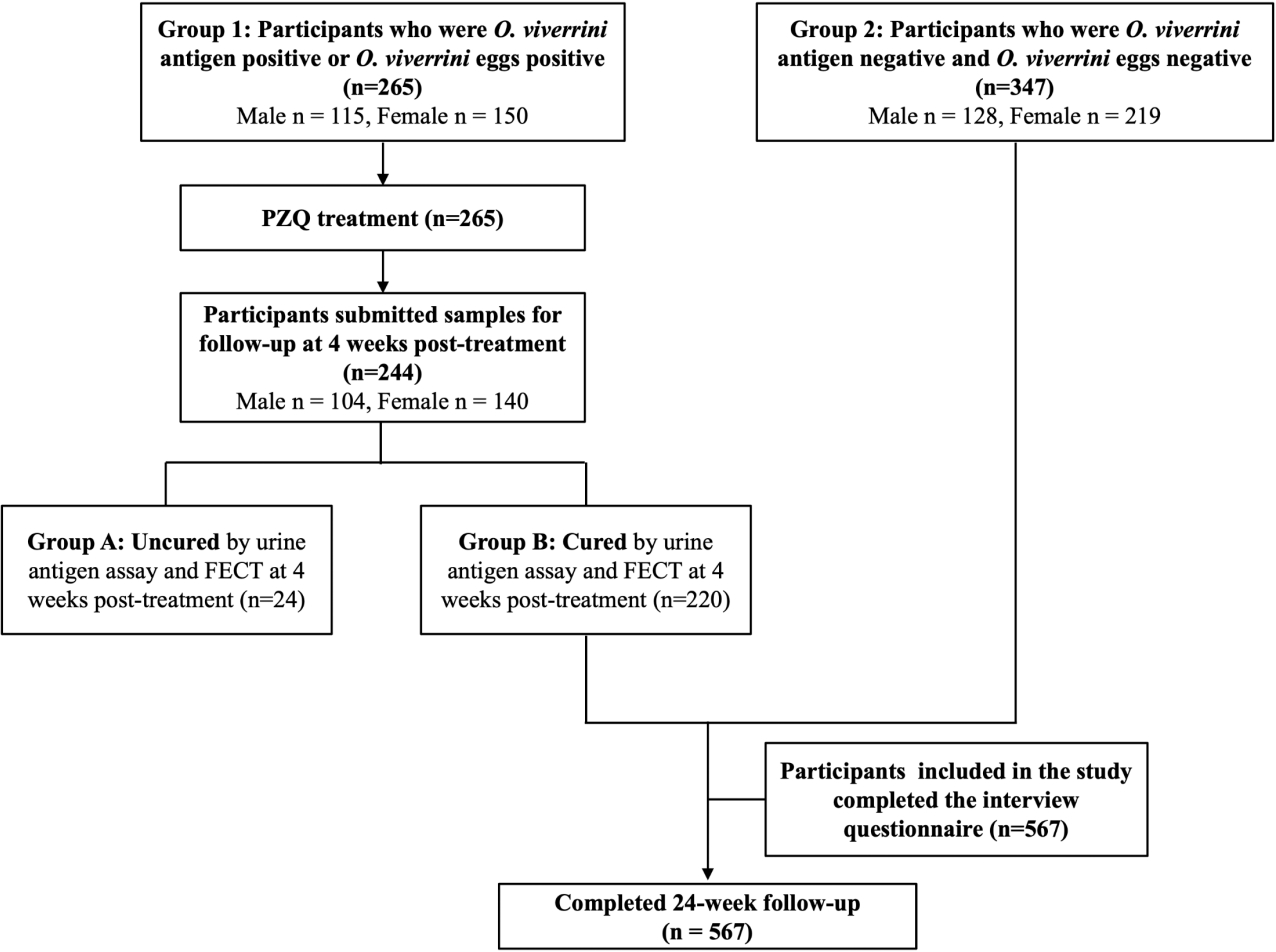
Flow diagram of study participants for the incidence and reinfection investigation, detailing sample size, types of diagnostic specimens collected, and the duration of follow-up assessments at 4- and 24-weeks. The diagnostic techniques for *O. viverrini* infection were FECT and urine antigen assays.

The research was a prospective study with multiple sampling at baseline and follow-up to assess incidence, reinfection rates, intensity of infection, and PZQ treatment. Participants were classified by baseline infection status based on the combined results of urine antigen ELISA and fecal examination. Group 1 comprised participants who tested positive for *O. viverrini* infection at baseline by either urine antigen ELISA or fecal examination and subsequently received PZQ treatment. These participants were reassessed at 4 weeks post-treatment to evaluate treatment response. Individuals who tested negative by urine antigen ELISA and fecal examination at the 4-week assessment were considered cured and were subsequently followed at week 24 to determine reinfection and infection intensity. Group 2 comprised participants who tested negative by both urine antigen ELISA and fecal examination at baseline screening. These participants were followed up at week 24 to determine the incidence of *O. viverrini* infection. Infection status of opisthorchiasis at follow-up was assessed using urine antigen ELISA, fecal examination, and questionnaire surveys (Fig 1).

### Sample size calculation

The sample size calculation was estimated using a single proportion formula: $n=\overset{\text{2}}{\underset{\alpha }{Z}}\left[p(\text{1}-p)\right]/d^{\text{2}}$ where *p* represents the estimated the prevalence of *O. viverrini*, based on prior data indicating a prevalence of 32.6% [26], *Z* is the level of confidence (1.96), and the acceptable margin of error (*d*) was set at 5% [27]; this gave a minimum sample size of 540 individuals. To account for possible non-response or participant dropout, an estimated attrition rate of 5% was applied. After this modification, 567 participants were ultimately determined to be the required sample size to maintain statistical power.

### Clinical sample collection

Clean plastic containers labeled with identifying numbers were distributed to project participants to collect samples, including fecal samples (approximately 10 grams) and first-morning, midstream urine samples (approximately 10 mL). Fecal samples were stored in containers at ambient temperature. In contrast, urine samples were stored in a chilled, insulated box and transported from the study site to the laboratory at Khon Kaen University within 1 day of collection. At the laboratory, fecal samples were weighed, fixed in 10% formalin, and processed for parasite examination using the formalin-ethyl acetate concentration technique (FECT). Urine samples were centrifuged at 988 × *g* at 4 °C for 15 minutes, and the supernatants were aliquoted and stored at −20 °C until use in the urine assay.

### Fecal examination by FECT

The quantitative FECT procedure was performed as previously described [26]. Briefly, fecal samples were homogenized, 2 grams of fresh feces were weighed, diluted in 7 mL of 10% formalin solution, thoroughly shaken, and then strained through gauze. Three milliliters of ethyl acetate were added to the mixture to extract fat from the feces. After vigorous shaking and centrifugation at 1,455 × *g* for 5 minutes, the supernatant was discarded, and the remaining material was resuspended in 10% formalin. After processing, the specimens were examined under a microscope, and the number of *O. viverrini* eggs was recorded in three drops from the total suspension by two different readers. The intensity of infection was calculated as the number of eggs counted per drop examined, divided by 2 (grams of stool), and multiplied by the total number of drops (volume) of the fecal suspension. Discrimination between eggs of *O. viverrini* and minute intestinal fluke (MIF), such as *Phaneropsolus bonnei* or *Prostodendrium molenkapi*, was performed based on morphological characteristics as previously described [28,29].

### Procedure for urine antigen detection method by monoclonal antibody-based enzyme-linked immunosorbent assay (mAb-ELISA)

The protocol for the *O. viverrini* antigen assay in urine using a monoclonal antibody-based ELISA has been described previously [30]. Briefly, flat-bottomed 96-well microtitre plates (Nunc, Roskilde, Denmark) were coated with 5 µg/mL of the monoclonal antibody (specific for the *O. viverrini*, clone KKU 505) and incubated overnight at 4°C. The next day, plates were washed three times with a buffer containing 0.05% Tween-20 in phosphate-buffered saline (PBST; pH 7.4), and uncoated sites were blocked with 5% dried skimmed milk in PBST. The plates were then incubated at 37 °C for 1 hour. Washing was repeated 3 times with PBST, and undiluted urine samples pre-treated with TCA (100 μL/well) were added to the wells in duplicate and incubated at 37 °C for 2 hours. The plates were washed 5 times with PBST, then IgG rabbit anti-crude *O. viverrini* antigen was added and incubated at 37 °C for 1 hour. After three washes, a 1:4,000 dilution of biotinylated goat anti-rabbit IgG (Invitrogen, Carlsbad, CA, USA) in PBST was added and incubated at 37°C for 1 hour. Thereafter, the plates were washed three times, and streptavidin horseradish peroxidase (HRP)-conjugate (GE Healthcare, Buckinghamshire, United Kingdom) (1:5000 dilution, 100 μL/well) was added. After incubation and washing, a substrate solution (o-phenylenediamine hydrochloride) (Sigma, St. Louis, MO, USA) (100 μL/well) was added, and plates were incubated for 20 min in the dark at room temperature. The reaction was stopped by the addition of 2M sulfuric acid (H_2_SO_4_), and the plates were read on an absorbance reader (Tecan, Grödig, Austria) at the optical density (OD) of 492 nm. The optical density values were converted to concentrations of *O. viverrini* antigens in urine using standard curves, then expressed as nanograms per milliliter [30,31]. A sample was considered positive when the antigen concentration in urine was > 32.94 ng/mL.

### Statistical analysis

Demographic information and responses to questions about potential risk factors from the questionnaires and laboratory data were entered into an Excel worksheet (Microsoft) and analyzed using SPSS 26 (IBM, Chicago, IL, USA). Baseline characteristics of the sample were presented as frequency numbers and percentages for categorical data. The continuous data were described using mean, median, and standard deviation (SD). The prevalence of *O. viverrini* infections was estimated overall and by gender and age group. Five age groups were established as follows: (i) ≤ 40 years, (ii) 41–50 years, (iii) 51–60 years, (iv) 61–70 years, and (v) > 70 years. Chi-squared tests were used to compare the prevalence of *O. viverrini* infection between age and gender. The incidence of infection was estimated as the cumulative proportion of new cases among individuals at risk over a 24-week (6-month) follow-up period, defined as the period from baseline assessment to the final survey. To facilitate comparison with previously published studies, incidence estimates were standardized and expressed as rates per 100 person-years, accounting for person-time at risk [18,32,33]. Reinfection rates among participants who received curative treatment were estimated over a 20-week (5-month) observation period, defined as the period from the post-treatment follow-up sampling at week 4 to the week 24 follow-up. Based on incidence, reinfection rates were standardized and reported per 100 person-years to ensure comparability. The infection intensity at pretreatment versus post-treatment, as measured by fecal egg count (EPG) and urine antigen levels, was compared using a paired t-test. In order to assess evidence of predisposition to infection, the correlations between fecal egg count and urine antigen concentration at baseline (week 0), 4 weeks after praziquantel treatment, and the 24-week follow-up were determined by Kendall’s τb correlation test. Risk factors for *O. viverrini* infection were reported as relative risk. The crude relative risk (RR) and adjusted relative risk (aRR) were calculated using a Poisson regression model. *P* values < 0.05 were considered statistically significant.

## Results

### Socioeconomic characteristics of the study participants

Of the 612 study participants, the majority (60.3%) were females, and 39.7% were males, and the average age was 56.6 years (standard deviation, SD 11.2). Most participants (85.0%) had a primary school education or less. The study population primarily consisted of farmers working in paddy fields (73.4%). Most participants (97.1%) had never been tested for *O. viverrini* infection, and 1.8% had a history of positive test results. Regarding lifestyle factors, alcohol consumption was reported by 10.3% of participants, while 8.7% were smokers. Notably, only 0.7% of participants reported a family history of liver cancer.

### Baseline prevalence of *O. viverrini* determined by urine assay and fecal examination

Based on ELISA-based urine antigen detection, the overall prevalence of *O. viverrini* was 41.0% (249 of 608). In contrast, fecal examination by FECT revealed an *O. viverrini* infection prevalence of 8.1% (42 of 522). The overall prevalence of parasitic infections, as determined by FECT, was presented in Table 1. The most common species was *O. viverrini*, with an overall prevalence of 8.1%. The second most common infection was *Strongyloides stercoralis,* with a prevalence of 3.3%, followed by minute intestinal flukes and echinostomes, each with a prevalence of 1.7%. Other infections were *Taenia* (1.0%) and *Trichuris trichiura* (0.2%).

**Table 1:** Prevalence of *O. viverrini* infection and other intestinal parasites in the studied community by FECT (n = 522).

Parasites	No. of positive	Prevalence (%)
*Opisthorchis viverrini*	42	8.1
*Strongyloides stercoralis*	17	3.3
Minute intestinal flukes	9	1.7
Echinostomes	9	1.7
*Taenia* sp.	5	1.0
*Trichuris trichiura*	1	0.2

### Age and gender prevalence and intensity of *O. viverrini*

**Fig 2:**
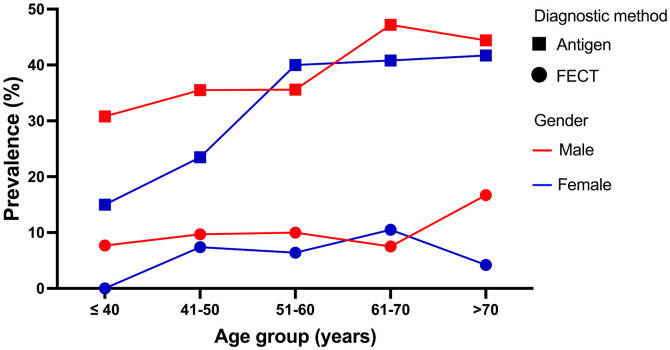
Age-sex-prevalence profiles of baseline *O. viverrini* infection determined by urine antigen assay and FECT.

The prevalence and distribution of *O. viverrini* by age and gender, determined by using FECT and the urine antigen assay on matched fecal and urine samples, are shown in Fig 2 (n = 518). The prevalence of *O. viverrini* increased significantly with age in both men and women, as determined by the urine antigen assay (χ^2^ = 16.8; p < 0.002). For the FECT, there was no association between age and infection prevalence (χ^2^ = 1.5; p > 0.05), with a peak at over 70 years for men and at 61–70 years for women.

### Incidence and reinfection rate of *O. viverrini* after PZQ treatment

Among participants who tested positive for *O. viverrini* infection at baseline by either urine antigen ELISA or fecal examination (Group 1, n = 244), 220 individuals (90.2%) were cured of opisthorchiasis at 4 weeks after praziquantel treatment. At the 24-week follow-up, corresponding to 20 weeks after confirmation of cure, 54 of these 220 participants tested positive by urine antigen ELISA, yielding a reinfection rate of 24.5%. Among participants who tested negative by both urine antigen ELISA and fecal examination at baseline (Group 2, n = 347), the incidence of *O. viverrini* infection at the 24-week follow-up was 32.3% based on urine antigen ELISA and 3.8% based on fecal examination (13 of 347 individuals). Overall, the combined reinfection rate and incidence detected by urine antigen ELISA or fecal examination at the 24-week follow-up was 30.7% (Table 2).

**Table 2:** Incidence and reinfection with *O. viverrini* were determined in subjects with baseline infection at 4 and 24 weeks after PZQ treatment using urinary antigen assays or fecal examinations. The subjects in group 1 were *O. viverrini*-positive at baseline, as determined by fecal examination or a urine antigen assay. The subjects in group 2 were urine antigen- and fecal egg-negative at baseline. Data shown are the number positive at baseline, number assessed, number positive (% of those assessed) determined by fecal examination and by urine antigen assay at intervals after treatment.

Sample groups	weeks 4		weeks 24			
	Assessed, n	Negative by the ELISA, n (%)	Assessed, n	Positive by the ELISA, n (%)	Positive by the FECT, n (%)	Positive by the ELISA or FECT, n (%)
1	244	220 (90.16)	220	54 (24.5)	5 (2.3)	57 (25.9)
2	N/A	N/A	347	112 (32.3)	13 (3.8)	117 (33.7)
Total			567	166 (29.3)	18 (3.2)	174 (30.7)

### Incidence and reinfection rates by sex and age using ELISA and FECT

Based on urine antigen detection by ELISA (Table 3A), reinfection rates were lower in males than in females, whereas incidence and combined rates were higher in males however, these differences were not statistically significant (p > 0.05). Incidence and combined rates increased with age, peaking in participants aged > 70 years (108.3 and 89.3 per 100 person-years, respectively), with a significant trend observed (χ² for trend = 9.791, p < 0.04). In contrast, reinfection rates showed no association with the age of the participants (p > 0.05). In the case of FECT, similar sex patterns were observed, with no significant differences between males and females (p > 0.05). No infections were detected in participants aged ≤ 40 years. Among older groups, incidence ranged from 5.8 to 10.3 per 100 person-years, and reinfection from 5.5 to 9.6 per 100 person-years, with no significant age-related trend (Table 3B).

**Table 3:** Patterns of sex- and age-related reinfection and incidence of *O. viverrini* infection, expressed as rate/100 person-years.

A. Based on urine antigen ELISA						
Factors	n	Re-infection (/100 person-years)	n	Incidence rate (/100 person-years)	n	Combined (/100 person-years)
**Gender**						
Male	91	57.1	128	71.9	219	66.7
Female	129	68.4	219	60.3	348	62.8
**Age groups (years)**						
≤ 40	8	97.4	28	35.7	36	46.8
41-50	31	50.4	78	59.0	109	57.0
51-60	95	68.3	138	59.4	233	62.5
61-70	59	57.3	79	75.9	138	69.1
> 70	27	67.3	24	108.3	51	89.3
**Total**	220	63.7	347	64.6	567	64.3
**B.** Based on fecal examination by FECT						
**Factors**	**n**	**Re-infection** **(/100 person-years)**	**n**	**Incidence rate (/100 person-years)**	**n**	**Combined (/100 person-years)**
**Gender**						
Male	91	5.7	128	4.7	219	5.1
Female	129	6.0	219	9.1	348	8.2
**Age groups (years)**						
≤ 40	8	0.0	28	0.0	36	0.00
41-50	31	0.0	78	10.3	109	7.9
51-60	95	5.5	138	5.8	233	5.7
61-70	59	8.8	79	10.1	138	9.6
> 70	27	9.6	24	8.3	51	8.9
**Total**	220	5.9	347	7.5	567	7.0

### Post-treatment changes in infection intensity

**Fig 3:**
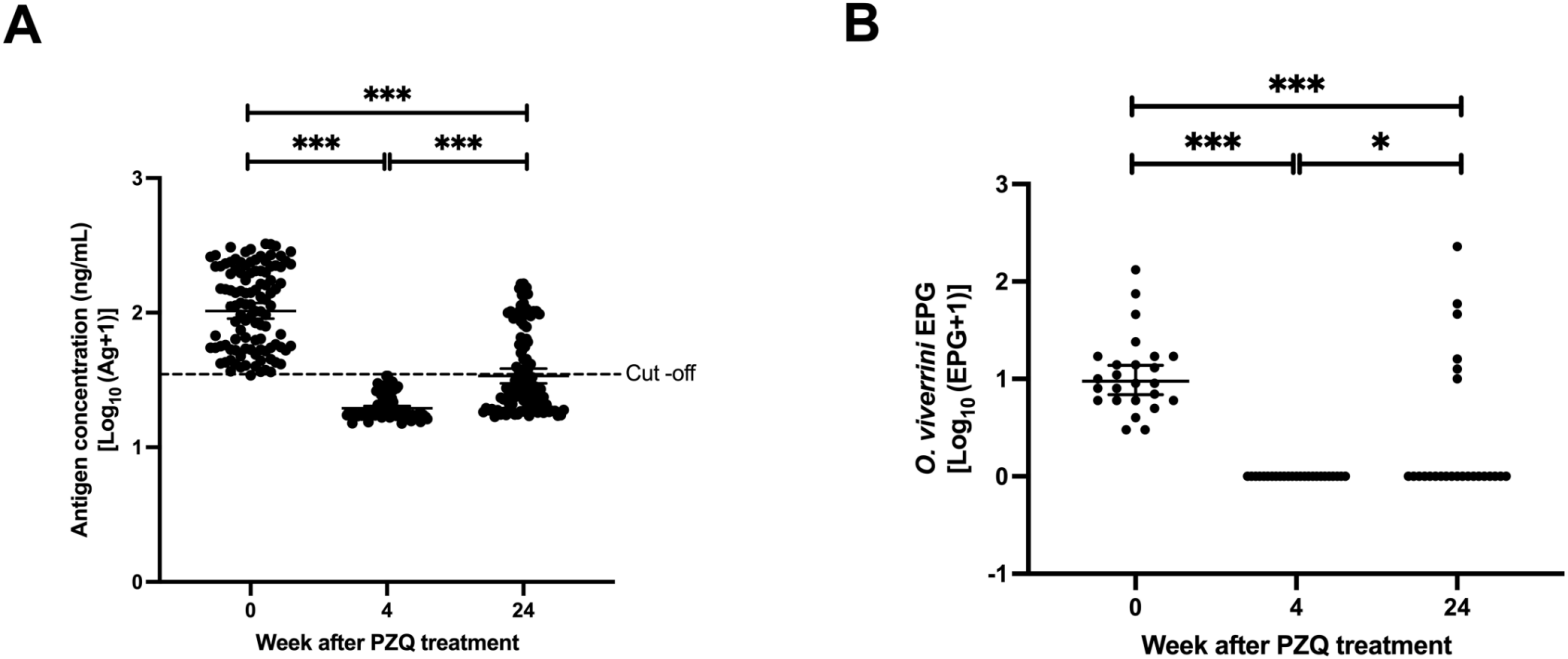
Time profiles for urine antigen concentrations (A) and intensity of *O. viverrini* infection measured by fecal egg counts (EPG) (B) in participants at baseline (week 0), 4 weeks after praziquantel treatment, and the 24-week follow-up. The data shown were observed values of urinary antigen concentration and EPG, and the solid lines were the means of log-transformed values. The horizontal dotted black line indicates the cutoff value for the urine antigen level. ***p < 0.001 and *p < 0.05.

Considering the intensity of infection, Fig 3A shows that the baseline antigen concentration (geometric mean [GM], 107.3 ng/mL [SD, 2.0]) became negative at 4 weeks after treatment and reappeared 20 weeks later (GM, 34.8 ng/mL [SD, 2.1]) (Kendall’s τb correlation; p < 0.001). In fecal egg counts, the baseline EPG (GM 9.7 [SD 2.7]) was cleared by PZQ treatment at 4 weeks post-treatment and reappeared due to reinfection at the 24-week follow-up. (Fig 3B). The correlation analysis of infection intensity (EPG) revealed a significant positive correlation between pre- and post-PZQ treatment (*R*^2^ = 0.144; p < 0.001; S1A Fig). No correlation was observed in urine antigen levels between pre- and post-treatment (p = 0.41) (S1B Fig).

### Risk factors associated with incidence and reinfection of *O. viverrini*

Based on a Poisson regression analysis with a positive diagnosis of opisthorchiasis by urine ELISA and/or FECT as the outcome variable, a history of treatment was associated with a higher risk of infection than among those who had not received prior anthelmintic treatment (aRR = 1.49, 95% CI: 1.04–2.14, p = 0.032). Moreover, eating raw fish in the previous 6 months was associated with an increased risk of infection compared with those who reported never eating raw fish (aRR = 7.52, 95% CI: 4.94–11.46, p < 0.001). Frequent raw fish consumption (>10 times per month) was significantly associated with an increased risk of infection compared with those who consumed it less frequently (aRR = 3.60; 95% CI: 2.85–4.56; p < 0.001). Other factors, including gender, age group, educational level, occupational status, smoking, and alcohol consumption, were not associated with *O. viverrini* infection over the 24-week study period (Table 4).

**Table 4:** The Poisson regression model, with relative risk (RR) and 95% confidence interval (95% CI), for the adjusted association of factors related to transmission of opisthorchiasis (combined incidence and/or reinfection) at the 24-week follow-up.

Factors	Number of *O. viverrini* infected persons	Person-years of follow-up	Incidence rate (/100 person-years)	RR	aRR	95%CI	p-value
Gender							
Females	70	109.5	63.9	1.02	1.09	0.75-1.57	0.653
Males	104	174.0	59.8	1.00	1.00		
Age groups (years)							
> 70	21	25.5	82.4	2.45	2.41	0.92-6.34	0.074
61-70	46	69.0	66.7	1.75	1.76	0.74-4.19	0.197
51-60	68	116.5	58.4	1.44	1.45	0.63-3.34	0.386
41-50	31	54.5	56.9	1.39	1.44	0.59-3.53	0.425
≤ 40	8	18.0	44.4	1.00	1.00		
Educational levels							
Primary and lower	151	240.5	62.8	1.25	1.01	0.58-1.78	0.962
Secondary and higher	23	43.0	53.5	1.00	1.00		
Occupational status							
Farmer	124	207.5	59.8	0.87	0.83	0.56-1.25	0.377
Non-farmer	50	76.0	65.8	1.00	1.00		
History of praziquantel treatment							
Once and over	92	129.0	71.3	1.53*	1.49	1.04-2.14	**0.032**
Never	82	154.5	53.1	1.00	1.00		
Smoking history							
Yes, current, or previous	18	25.0	72.0	1.30	1.26	0.65-2.44	0.495
No	156	258.5	60.3	1.00	1.00		
Alcohol consumption history							
Yes, current, or previous	16	28.0	57.1	0.89	0.90	0.48-1.68	0.733
No	158	255.5	61.8	1.00	1.00		
Eating uncooked fish in the previous 6 months							
Yes	135	129.0	104.7	7.60***	7.52	4.94-11.46	**<0.001**
No	39	154.5	25.2	1.00	1.00		
Frequency of raw fish consumption (times/months)							
>10	92	65.5	140.5	3.57***	3.60	2.85-4.56	**<0.001**
3-10	20	20.0	100.0	2.24***	2.20	1.57-3.09	**<0.001**
< 3	62	198.0	31.3	1.00	1.00		

The data were analyzed using a Poisson regression model, which yielded RR and aRR with 95% CI and p-values.

*, *** indicate RR with a significance level of P < 0.05, and P < 0.001, respectively.

### Raw fish consumption and behavioral risk factors on *O. viverrini* transmission

**Fig 4:**
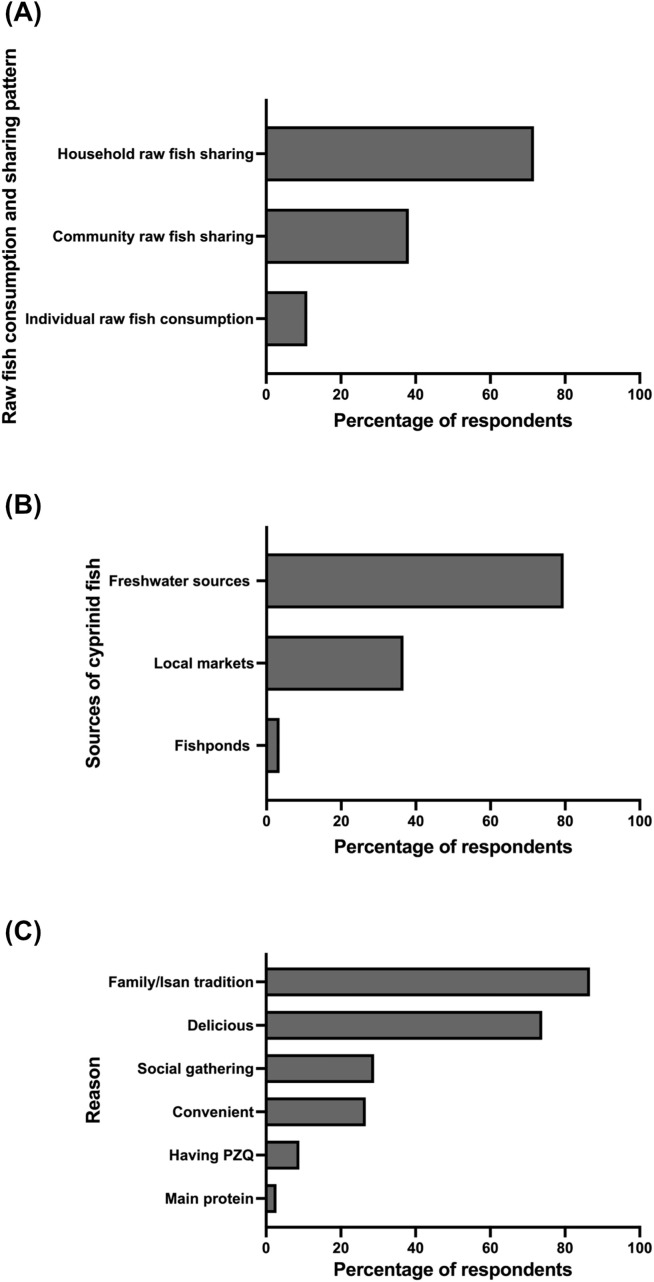
Data from project participants regarding the raw fish consumption and sharing patterns (A), sources of cyprinid fish (B), and motivations for consuming raw fish (C). Participants can select all reasons applicable, so the percentage values do not add up to 100%.

Among the 289 project participants who had consumed raw fish within the previous 6 months, 289 (100%) responded to the question on reasons for raw fish consumption and sharing patterns. Household-level raw fish sharing was the most predominant practice (71.7%), followed by community-level sharing (38.2%), whereas individual raw fish consumption was least frequent (11.0%) (Fig 4A). The majority of raw fish consumers obtained their fish from natural freshwater sources (79.6%), while a smaller proportion sourced fish from local markets (36.7%) and fishponds (3.5%) (Fig 4B). The primary reasons for consuming raw fish were family/ Isan tradition (86.7%) and taste preference (73.9%), followed by social gatherings (28.9%). Additional reasons for raw fish consumption included availability and convenience (26.7%), availability of PZQ (8.9%), and as a main protein source (2.8%), respectively (Fig 4C).

## Discussion

This study provides insights into the longitudinal patterns of opisthorchiasis through a prospective cohort study that assessed baseline prevalence, incidence, and reinfection detected at the 24-week follow-up, and includes an in-depth analysis of risk factors associated with *O. viverrini* infection in a rural community in northeastern Thailand. Despite past and current public health control programs, the finding of a high baseline prevalence of *O. viverrini* infection (41.0%) when assessed with a sensitive urine antigen test, compared with a fivefold lower prevalence determined by stool-based FECT (8.1%). In the follow-up study, the urine antigen assay detected higher incidence and reinfection rates than FECT. These results demonstrated the advantages of urine antigen testing in identifying *O. viverrini* infection and capturing longitudinal patterns of prevalence, incidence, and reinfection before and after praziquantel treatment [4,19,31,34,35].

The baseline prevalence of infection reflects cumulative exposure to infection over time in opisthorchiasis, and the age of the participants represents the duration of exposure to infection. To understand how the prevalence of infection rebounds after chemotherapeutic control, the current study measured the incidence rate (i.e., infection rate) in *O. viverrini*-negative individuals at baseline and the reinfection rate among individuals cured by treatment. Within the cohort in Northeast Thailand, the incidence of opisthorchiasis based on urine ELISA was 32.3%, which was tenfold higher than that detected by FECT (3.8%). Concurrently, the reinfection rate of opisthorchiasis over the same duration was 24.5% by urine ELISA and was almost tenfold higher than that by FECT (2.3%) over a 24-week duration. These results once again confirmed the superior sensitivity of urine antigen detection over fecal examination, as previously reported [30,31]. We previously reported the use of urine antigen ELISA to measure the reinfection rate of opisthorchiasis over a longer period of 48 weeks post-treatment [19]. This is the first time antigen detection in urine has been used to assess the incidence and reinfection rate of opisthorchiasis over a duration of 24-week follow-up. Evidence from experimental studies in laboratory animals showed positive antigen detection was found as early as 1–3 weeks post-infection when the worms were immature [19]. It remains to be seen whether incidence and reinfection in humans can be measured over a short period (i.e., < 6 months) to closely monitor transmission in humans.

The majority of the study cohort predominantly comprised middle-aged to older agricultural workers with low levels of formal education. Factors associated with a higher risk of *O. viverrini* infection included prolonged exposure to endemic areas and customary eating habits. The greater odds of *O. viverrini* infection in both sexes across older age groups, especially in individuals over 50 years of age, may indicate a lifetime accumulation of exposure and chronicity [36]. This highlights the need to screen older people by abdominal ultrasonography for potential chronic infection, as there is a prolonged risk of the development of CCA, a lethal bile duct cancer [37].

Fecal egg detection represents an imperfect gold standard, as egg excretion can be intermittent and often falls below detectable levels in individuals with low worm burdens in opisthorchiasis [3,38,39]. The biological interpretation of urine antigen positivity indicates active infection of *O. viverrini* as observed in animal and human studies [34,35]. However, the positive urine antigen ELISA result may be associated with other possibilities such as residual circulating parasite antigens that persist following treatment, delayed antigen clearance, or potentially very early-stage infection before the onset of detectable egg shedding [40,41]. Therefore, the higher prevalence, incidence, and reinfection rates observed by urine antigen ELISA than those by FECT reflect its greater sensitivity rather than reduced specificity. The antigen levels became negative after 4–6 weeks of praziquantel treatment [19,42]. Incorporating the urine antigen assay alongside the FECT for screening for opisthorchiasis and follow-up of patients after anthelminthic treatment may mitigate the drawbacks of the FECT and provide a more comprehensive assessment of opisthorchiasis infection dynamics.

The analysis of factors associated with incidence and reinfection identified several behavioral risk factors, including a history of praziquantel treatment, recent consumption of raw fish, and frequent consumption of raw fish (>10 times per month). The present study found that participants with a history of praziquantel treatment had a significantly higher likelihood of *O. viverrini* infection compared with those who had never received treatment. Individuals who reported one or more prior praziquantel treatments had a 1.49-fold higher risk of infection, consistent with findings from previous studies [43]. In endemic communities in Northeast Thailand, the consumption of raw or undercooked cyprinid fish remains common due to longstanding cultural dietary practices. Consequently, individuals who previously received praziquantel treatment may continue engaging in behaviors that place them at risk of reinfection [43,44]. Frequent praziquantel use may therefore serve as an indicator of persistent high-risk behavior and ongoing environmental exposure rather than protection against future infection. Similarly, consumption of raw fish within the past six months was the strongest predictor of disease, underscoring the impact of continued traditional food practices in sustaining transmission cycles. A significant finding was the elevated incidence of at-risk behavior. Participants reported consuming raw or fermented fish dishes, notably the spicy fermented fish dip, a practice deeply rooted in cultural and community traditions. Similar research has consistently highlighted the strong influence of social norms and familial practices on raw-fish consumption in endemic regions [45,46]. The current findings indicate a robust, statistically significant correlation between regular consumption of raw or undercooked fish and infection risk of infection. These findings align with the current literature, indicating that repeated exposure significantly increases the likelihood of ingesting viable infective stages, the principal pathway for *O. viverrini* transmission in the region [3,38].

Seasonal conditions influence the transmission of *O. viverrini* as the infection status of intermediate hosts, with previous studies reporting higher metacercarial burdens in cyprinid fish during the late rainy season and winter months [47,48]. Increased rainfall and flooding can facilitate the dispersal of egg-contaminated feces into aquatic environments, expand suitable habitats for *Bithynia* snails, and enhance the distribution of infected fish, thereby increasing opportunities for parasite transmission [49,50]. This study was conducted from April to November 2024, partially covering the peak of transmission in the rainy to winter season in Northeastern Thailand. Whether additional follow-up to cover the entire winter season (November to February) will affect the rate of parasite transmission (incidence and reinfection) remains to be investigated.

Sociocultural factors may further contribute to transmission. In the present study, the sharing of meals containing raw fish was commonly reported, reflecting a widespread cultural practice in rural Northeastern Thailand and the Lao PDR. This practice may promote clustered and repeated exposure to infective metacercariae among household members and community groups, thereby sustaining transmission within endemic populations. This also highlights the importance of collective sociocultural factors in the maintenance of endemicity, which is otherwise mainly attributed to individual behaviors. Our results corroborate previous epidemiological studies in endemic areas, which reported a high frequency of raw fish consumption as the strongest predictor of infection [51–54]. Thus, culturally appropriate behavioral change interventions addressing high rates of raw fish consumption and communal fish sharing, together with health education campaigns and chemotherapy, are required for long-term control. Geographically, the study area in our study is located near the Lampao Reservoir in Kalasin Province, which is part of the endemic wetlands of *O. viverrini* in Northeast Thailand [54,55]. The combination of favorable environmental conditions and persistent sociocultural practices likely contributes to the continued endemicity observed in this region.

There are limitations in this study that need to be acknowledged for further study. First, its single-community design and reliance on self-reported behaviors may have been affected by social desirability bias. Second, a single fecal sample was collected for FECT examination, which may lead to underdiagnosis of parasitic infection compared with a three-day examination, which may improve test results [56,57]. Third, because FECT cannot distinguish MIF and *O. viverrini eggs* in both baseline screening and follow-up examinations, PCR confirmation should be included in future studies [58]. Fourth, to capture patterns of incidence and reinfection rates, future research should focus on using urinary antigen detection across diverse transmission settings in different regions of Thailand, with larger sample sizes and multiple follow-up sampling periods. Lastly, a rapid point-of-care test for opisthorchiasis screening is more suitable than ELISA [40], which was not available at the time of this study.

## Conclusion

The results of this study highlighted both the reinfection rate and the incidence of opisthorchiasis. Based on urine ELISA, the baseline prevalence, reinfection, and incidence rates were several-fold higher than those obtained by fecal examination. The observation of rebounded prevalence indicates ongoing transmission of opisthorchiasis; thus, continued fish consumption persists despite a marked short-term decline in infection following praziquantel administration. Therefore, further research on changes in eating behavior and the barriers to sustaining such changes among high-risk groups with opisthorchiasis is required.

## Supporting information

S1 Fig Correlations between pre- and post-treatment intensity of *Opisthorchis viverrini* infection at 24-week follow-up, as evaluated by the formalin-ethyl acetate concentration technique (FECT) and the urine antigen assay. Panels A and B demonstrate the relationships between pre- and post-treatment *O. viverrini* egg counts (A) and urine antigen levels (B) among participants with reinfection.
(TIFF)
