# Supplementary figures and images for: Longitudinal changes and risk factors of Opisthorchis viverrini infection after selective praziquantel treatment: evidence from urine antigen assay and fecal examination in an endemic community in Northeast Thailand
Source: PLoS One. 2026 Jul 6;21(7):e0352854. doi: 10.1371/journal.pone.0352854 (PMC13336184; doi:10.1371/journal.pone.0352854)

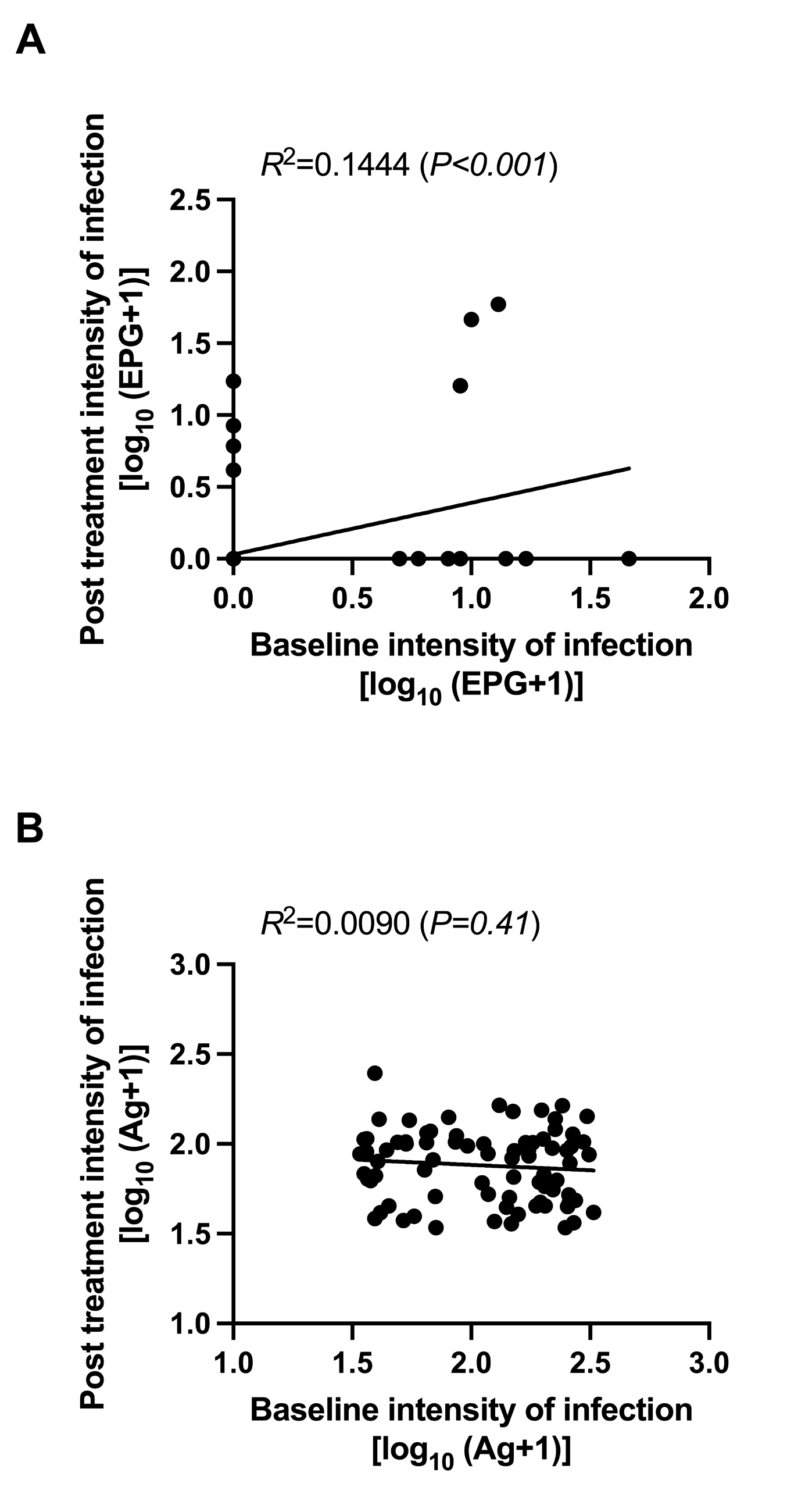

Supplement: S1 Fig — Panels A and B demonstrate the relationships between pre- and post-treatment O. viverrini egg counts (A) and urine antigen levels (B) among participants with reinfection. (TIFF) [file pone.0352854.s001.tiff]
