# Supplementary material for: Longitudinal changes and risk factors of Opisthorchis viverrini infection after selective praziquantel treatment: evidence from urine antigen assay and fecal examination in an endemic community in Northeast Thailand
Source: PLoS One. 2026 Jul 6;21(7):e0352854. doi: 10.1371/journal.pone.0352854 (PMC13336184; doi:10.1371/journal.pone.0352854)
Supplement: Attachment — Submitted filename: Response to reviewers_R2.docx [file pone.0352854.s004.docx]

**Response to Editor**

**Journal Requirements:**

If applicable, we recommend that you deposit your laboratory protocols in protocols.io to enhance the reproducibility of your results. Protocols.io assigns your protocol its own identifier (DOI) so that it can be cited independently in the future. For instructions see: https://journals.plos.org/plosone/s/submission-guidelines#loc-laboratory-protocols. Additionally, PLOS ONE offers an option for publishing peer-reviewed Lab Protocol articles, which describe protocols hosted on protocols.io. Read more information on sharing protocols at https://plos.org/protocols?utm_medium=editorial-email&utm_source=authorletters&utm_campaign=protocols.

Response 1:

Thank you for this recommendation. The laboratory protocols used in this study, including the formalin-ethyl acetate concentration technique (FECT) for fecal examination and the urine antigen ELISA for detecting *Opisthorchis viverrini* infection, are well-established methods that have been described in detail and previously published. Appropriate references to these protocols have been provided in the Methods section of the manuscript.

Specifically, the FECT procedure was performed according to the previously published protocol described by Kopolrat et al. (2022), while the urine antigen ELISA was conducted following the methods reported by Worasith et al. (2015) and Worasith et al. (2019).

As the corresponding author, your ORCID iD is verified in the submission system and will appear in the published article. PLOS supports the use of ORCID, and we encourage all coauthors to register for an ORCID iD and use it as well. Please encourage your coauthors to verify their ORCID iD within the submission system before final acceptance, as unverified ORCID iDs will not appear in the published article. Only the individual author can complete the verification step; PLOS staff cannot verify ORCID iDs on behalf of authors.

Response 2:

Thank you for this information. We will encourage all coauthors to register for and verify their ORCID IDs within the submission system prior to final acceptance so that their verified ORCID IDs can be included in the published article.

If the reviewer comments include a recommendation to cite specific previously published works, please review and evaluate these publications to determine whether they are relevant and should be cited. There is no requirement to cite these works unless the editor has indicated otherwise.

Response 3:

Thank you for this guidance. We carefully reviewed all reviewer comments and confirmed that no specific previously published works were recommended for citation. Therefore, no additional references were added in response to this comment.

Please review your reference list to ensure that it is complete and correct. If you have cited papers that have been retracted, please include the rationale for doing so in the manuscript text, or remove these references and replace them with relevant current references. Any changes to the reference list should be mentioned in the rebuttal letter that accompanies your revised manuscript. If you need to cite a retracted article, indicate the article’s retracted status in the References list and also include a citation and full reference for the retraction notice.

Response 4:

Thank you for this guidance. We carefully reviewed all reviewer comments and confirmed that no specific previously published works were recommended for citation. Therefore, no additional references were added in response to this comment.

**#Respond to the Editor (highlighted in yellow)**

First, please verify the source and license of the underlying geospatial data used in Fig. 1. The response indicates that the figure was created in QGIS, but QGIS is the software used to prepare the map and does not by itself establish that the underlying map layers, boundaries, or other geospatial data are compatible with publication under the CC BY 4.0 license. Please add the actual data source(s) for the map layers to the figure caption and confirm that these data can be redistributed under the journal’s license.

Response 1:

Thank you for this comment. We confirm that QGIS was used solely as the software to prepare Figure 1. The underlying geospatial data consisted of administrative boundary shapefiles obtained from the United Nations Office for the Coordination of Humanitarian Affairs (UN-OCHA) Humanitarian Data Exchange (HDX) geoBoundaries dataset for Thailand. We have revised the figure caption to include the source of the geospatial data and confirmed that these data can be redistributed under the applicable open-data license in accordance with the journal's requirements. (Materials and methods section, lines 108-113)

Second, please harmonize the ethics and consent wording between the manuscript and the submission form. The submission ethics statement indicates that only participants who provided written informed consent were included, whereas the manuscript states that adults provided written informed consent and that parents or legal guardians provided verbal consent for participants aged 15–17 years. Please ensure that the wording is consistent and that the reported form of consent for minors matches the ethics approval.

Response 2:

Thank you for highlighting this inconsistency. We have reviewed the ethics approval documents and revised the manuscript and submission information to ensure consistency in the description of the informed consent procedures. The revised wording now states that written informed consent was obtained from all adult participants and that written informed consent was obtained from the parents or legal guardians of participants aged 15-17 years prior to enrollment in the study. The ethics and consent statements are now fully aligned throughout the manuscript and submission materials. (Materials and methods section, lines 117-120)
