# Supplementary material for: Longitudinal changes and risk factors of Opisthorchis viverrini infection after selective praziquantel treatment: evidence from urine antigen assay and fecal examination in an endemic community in Northeast Thailand
Source: PLoS One. 2026 Jul 6;21(7):e0352854. doi: 10.1371/journal.pone.0352854 (PMC13336184; doi:10.1371/journal.pone.0352854)
Supplement: Attachment — Submitted filename: Response to reviewer_R1.docx [file pone.0352854.s003.docx]

**Response to Editor and Reviewer**

**#1 Respond to the Editor**

1. Please ensure that your manuscript meets PLOS ONE's style requirements, including those for file naming. The PLOS ONE style templates can be found at

https://journals.plos.org/plosone/s/file?id=wjVg/PLOSOne_formatting_sample_main_body.pdf and 
https://journals.plos.org/plosone/s/file?id=ba62/PLOSOne_formatting_sample_title_authors_affiliations.pdf.

**Response 1**:

Thank you for bringing this to our attention. We have carefully reviewed the manuscript and revised it to comply with PLOS ONE formatting and style requirements, including manuscript structure, section headings, references, tables, figures, and file-naming conventions, in accordance with the journal's guidelines and templates.

2. Thank you for stating the following financial disclosure:

“The Kasetsart University Research and Development Institute, KURDI (Grant number YF(KU)53.66), and Fluke-Free Thailand, National Research Council of Thailand. “

Please state what role the funders took in the study.  If the funders had no role, please state: "The funders had no role in study design, data collection and analysis, decision to publish, or preparation of the manuscript."

If this statement is not correct you must amend it as needed.

Please include this amended Role of Funder statement in your cover letter; we will change the online submission form on your behalf.

**Response 2**:

Thank you for this comment. We confirm that the funders had no role in study design, data collection and analysis, decision to publish, or preparation of the manuscript. We have revised the Financial Disclosure statement accordingly.

3. In this instance it seems there may be acceptable restrictions in place that prevent the public sharing of your minimal data. However, in line with our goal of ensuring long-term data availability to all interested researchers, PLOS’ Data Policy states that authors cannot be the sole named individuals responsible for ensuring data access (http://journals.plos.org/plosone/s/data-availability#loc-acceptable-data-sharing-methods).

Data requests to a non-author institutional point of contact, such as a data access or ethics committee, helps guarantee long term stability and availability of data. Providing interested researchers with a durable point of contact ensures data will be accessible even if an author changes email addresses, institutions, or becomes unavailable to answer requests.

Before we proceed with your manuscript, please also provide non-author contact information (phone/email/hyperlink) for a data access committee, ethics committee, or other institutional body to which data requests may be sent. If no institutional body is available to respond to requests for your minimal data, please consider if there any institutional representatives who did not collaborate in the study, and are not listed as authors on the manuscript, who would be able to hold the data and respond to external requests for data access? If so, please provide their contact information (i.e., email address). Please also provide details on how you will ensure persistent or long-term data storage and availability.

**Response 3**:

Thank you for this comment. All relevant data underlying the findings of this study are provided within the manuscript and its Supporting Information files.

4. Please be informed that funding information should not appear in the Acknowledgments section or other areas of your manuscript. We will only publish funding information present in the Funding Statement section of the online submission form. Please remove any funding-related text from the manuscript.

**Response 4**:

We have reviewed the manuscript to ensure compliance with PLOS ONE's funding disclosure requirements. The Acknowledgments section contains only acknowledgments of study participants and collaborating institutions and does not include any funding-related information. Funding details are reported exclusively in the Funding Statement section of the online submission form.

5. We note that Figure 1 in your submission contain [map/satellite] images which may be copyrighted. All PLOS content is published under the Creative Commons Attribution License (CC BY 4.0), which means that the manuscript, images, and Supporting Information files will be freely available online, and any third party is permitted to access, download, copy, distribute, and use these materials in any way, even commercially, with proper attribution. For these reasons, we cannot publish previously copyrighted maps or satellite images created using proprietary data, such as Google software (Google Maps, Street View, and Earth). For more information, see our copyright guidelines: http://journals.plos.org/plosone/s/licenses-and-copyright.
 
We require you to either (1) present written permission from the copyright holder to publish these figures specifically under the CC BY 4.0 license, or (2) remove the figures from your submission:
 
a. You may seek permission from the original copyright holder of Figure 1 to publish the content specifically under the CC BY 4.0 license.  
 
We recommend that you contact the original copyright holder with the Content Permission Form (http://journals.plos.org/plosone/s/file?id=7c09/content-permission-form.pdf) and the following text:
“I request permission for the open-access journal PLOS ONE to publish XXX under the Creative Commons Attribution License (CCAL) CC BY 4.0 (http://creativecommons.org/licenses/by/4.0/). Please be aware that this license allows unrestricted use and distribution, even commercially, by third parties. Please reply and provide explicit written permission to publish XXX under a CC BY license and complete the attached form.”
 
Please upload the completed Content Permission Form or other proof of granted permissions as an "Other" file with your submission.
 
In the figure caption of the copyrighted figure, please include the following text: “Reprinted from [ref] under a CC BY license, with permission from [name of publisher], original copyright [original copyright year].”
 
b. If you are unable to obtain permission from the original copyright holder to publish these figures under the CC BY 4.0 license or if the copyright holder’s requirements are incompatible with the CC BY 4.0 license, please either i) remove the figure or ii) supply a replacement figure that complies with the CC BY 4.0 license. Please check copyright information on all replacement figures and update the figure caption with source information. If applicable, please specify in the figure caption text when a figure is similar but not identical to the original image and is therefore for illustrative purposes only.
The following resources for replacing copyrighted map figures may be helpful:
 
USGS National Map Viewer (public domain): http://viewer.nationalmap.gov/viewer/
The Gateway to Astronaut Photography of Earth (public domain): http://eol.jsc.nasa.gov/sseop/clickmap/
Maps at the CIA (public domain): https://www.cia.gov/library/publications/the-world-factbook/index.html and https://www.cia.gov/library/publications/cia-maps-publications/index.html
NASA Earth Observatory (public domain): http://earthobservatory.nasa.gov/
Landsat: http://landsat.visibleearth.nasa.gov/
USGS EROS (Earth Resources Observatory and Science (EROS) Center) (public domain): http://eros.usgs.gov/#
Natural Earth (public domain): http://www.naturalearthdata.com/

**Response 5**:

Thank you for this comment. We have reviewed Figure 1 to ensure compliance with the PLOS ONE copyright and licensing requirements. Figure 1 was created by the authors using QGIS software, which is distributed under the Creative Commons Attribution 4.0 International License. No copyrighted Google Maps, Google Earth, or other proprietary map or satellite imagery was used in the preparation of this figure. We have revised the manuscript to clarify the source of the map and confirm that the figure complies with the CC BY 4.0 licensing requirements.

6. If the reviewer comments include a recommendation to cite specific previously published works, please review and evaluate these publications to determine whether they are relevant and should be cited. There is no requirement to cite these works unless the editor has indicated otherwise. 

**Response 6**:

Thank you for this guidance. We confirm that we have reviewed all reviewer comments and note that no specific additional references were requested. We have ensured that the manuscript cites the most relevant literature to support the study background, methods, and interpretation of findings.

**Respond to the Reviewer #1 (highlighted in blue)**

Reviewer #1: The manuscript presents a strong and well-designed epidemiological study investigating the transmission dynamics of Opisthorchis viverrini infection in an endemic region of Northeast Thailand. The prospective cohort design, the analysis of both incidence and reinfection, and the integration of urine antigen detection together provide important and highly interesting findings with clear public health relevance. In particular, the application of urinary antigen ELISA for monitoring transmission dynamics represents an important contribution to the field and highlights the potential advantages of non-invasive diagnostic approaches in opisthorchiasis surveillance. The manuscript is generally well written, scientifically sound, and supported by robust epidemiological data. The discussion is balanced and appropriately cautious. However, I would ask the authors to further discuss several important methodological and interpretational issues before publication.

**Comments 1:** FECT as an imperfect reference standard. This is probably the most important methodological issue of the manuscript. The diagnostic performance of the urine ELISA is evaluated against FECT, although FECT itself has relatively low sensitivity and cannot reliably distinguish *O. viverrini* eggs from minute intestinal flukes. Therefore, sensitivity, specificity, kappa values, and false-positive calculations may be distorted by the limitations of the reference standard itself. The authors partially acknowledge this issue; however, I recommend emphasizing this point more clearly in the Discussion section. In particular, the following statement or a similar interpretation should be considered: “Because FECT is an imperfect reference standard, the reported diagnostic performance metrics of urine ELISA should be interpreted cautiously.”

**Response 1**:

Thank you for this helpful suggestion. Accordingly, we have revised the Discussion section to explicitly state that FECT is an imperfect reference standard and may fail to detect low-intensity infections, and hence the reported diagnostic performance measures of the urine ELISA should therefore be interpreted with caution (Discussion section, lines 407-410).

**Comments 2:** Interpretation of ELISA-positive cases: The manuscript would benefit from a more detailed discussion regarding the biological interpretation of urine antigen positivity. It is not entirely certain that every ELISA-positive individual represents an active egg-producing infection. Urinary antigen positivity may also reflect residual circulating antigens, delayed antigen clearance after treatment, or possibly very early-stage infection before detectable egg shedding. This important immunological and diagnostic aspect deserves further discussion.

**Response 2**:

Thank you for this insightful comment in adding more reasons and interpretations of urine antigen positivity. We have revised the Discussion section to acknowledge that a positive urine ELISA result may not entirely indicate an active egg-producing infection and may reflect residual circulating antigens, delayed antigen clearance following treatment, or early-stage infection prior to detectable egg shedding. We have also noted that these situations may contribute to discordance between urine antigen detection and fecal examination in the longitudinal study. (Discussion section, lines 410-418).

**Comments 3:** Reinfection versus treatment failure: The manuscript interprets positivity at 24 weeks mainly as reinfection. However, it remains unclear whether all cases truly represent reinfection or whether some individuals may have experienced partial treatment failure, persistent low-level infection, or subcurative praziquantel response. I recommend that the authors discuss this distinction more thoroughly, as it may influence the interpretation of reinfection dynamics.

**Response 3**:

Thank you for this important comment. In our study, reinfection was assessed only among the 220 participants who were antigen- or egg-negative at 4 weeks post-treatment with praziquantel. Thus, the reinfection rate was calculated among individuals considered cured according to the study's diagnostic criteria. Indeed, 166 of 220 individuals were consistently antigen-negative at both the 4- and 24-week follow-ups, suggesting true curative treatment. Nevertheless, we acknowledge that a negative result at week 4 does not completely exclude the possibility of residual low-intensity infection below the detection limit of the diagnostic methods used. Therefore, some positive cases detected at week 24 may have represented persistent low-level infection, partial treatment failure, or subcurative response to praziquantel rather than true reinfection alone. Because no additional diagnostic assessments were conducted between weeks 4 and 24, these possibilities cannot be completely ruled out. We have revised the Discussion section to acknowledge this limitation and to interpret reinfection estimates with appropriate caution (Lines 416-419).

**Comments 4:** Increased risk associated with previous praziquantel treatment: The finding that prior praziquantel treatment is associated with higher infection risk is epidemiologically very interesting. However, this association may be strongly confounded. It is possible that individuals with repeated praziquantel treatments are simply those with the highest long-term exposure risk and most persistent behavioural risk factors. Therefore, previous praziquantel treatment history may function more as a marker of repeated exposure rather than an independent biological risk factor. This interpretation should be further elaborated in the Discussion.

**Response 4**:

Thank you for this insightful comment. We agree that the observed association between prior praziquantel treatment and *O. viverrini* infection suggests potential reinfection especially in individuals with a history of repeated praziquantel treatment due to persistent exposure to infection due to continued consumption of raw fish. Therefore, a history of previous praziquantel treatment may serve as a marker of repeated exposure and reinfection risk rather than an independent biological risk factor. We have revised the Discussion section to clarify this interpretation and to acknowledge the potential for confounding by unmeasured behavioral and environmental factors (Lines 426-432).

**Comments 5:** Duration of follow-up and potential seasonal effects: The 6-month follow-up period is appropriate for evaluating short-term epidemiological dynamics; however, it may not fully capture seasonal transmission patterns. I would ask the authors to comment on whether the study period coincided with specific environmental or sociocultural factors in the region, such as fishing seasons, rainy season-related ecological changes, national or local festivals involving traditional raw fish consumption. These factors could potentially influence transmission intensity and reinfection rates and would strengthen the epidemiological interpretation of the findings.

Overall, this is an important and valuable study with strong epidemiological content and highly relevant findings. After addressing the points above, I believe the manuscript would make a meaningful contribution to the field.

**Response 5**:

Thank you for this valuable suggestion. We agree that seasonal and sociocultural factors may influence *O. viverrini* transmission and reinfection patterns. The study was conducted from April to November 2024, covering much of the rainy season in Northeastern Thailand (May-October), when freshwater ecosystems are most productive, and fishing activities are common. This period may increase access to and consumption of cyprinid fish, which serve as the second intermediate host of *O. viverrini*.

We have added texts in the Discussion section concerning the potential contributors to the incidence and reinfection rates observed in the study in lines 443-452.

**Respond to the Reviewer #2 (highlighted in green)**

Reviewer #2: Dear authors. I've read this manuscript with interest. Congratulations. I was surprised to see that during the 24 w study period, the incidense was higher in those negative at baseline (hence not infected during the previous years) than the reinfection rate in those positive at baseline. But that is what the numbers show. The manuscript is well written in fluent language.

Major:
*The main problem that I find is the relation between the two methods; urinary ELISA and FECT. Using the ELISA it is a 5-10-fold (!) increase in prevalence/incidense/reinfection rate. It is well known (as written in the manuscript) that FECT is an imperfect 'gold standard' with both false positives and especially false negatives. It is almost comparing apples and oranges and make the section The diagnostic accuracy difficult (and unnecessary??). In line 324 it states ELISA has sensitivity 61.9%, which is not so good when the number of positive ELISA is so much higher than FECT (due to false positive 'gold standard'/FECT?).

Table 4 mix just one technical test quantification (sensitivity) and one prevalence dependent diagnostic predictive value (NPV).

In line 328 it says: 'The positive urine ELISA results among fecal egg-negative individuals (i.e. false positives) ranged from 0.1% to 1.6%.' How do you calculate those numbers? There is a 5-10-fold higher number of ELISA positives than FECT positives.

I suggest the diagnostic accuracy section is rewritten for clarity. As the study is not really designed to validate the test properties of the two diagnostic methods, you may alternativly consider if the section can be omitted and the difference in test properties be discussed in Discussion, based on findings from previous studies. Especially interesting is the assumed specificity of ELISA (many false positives?)

**Response 1**:

Thank you for this valuable comment. We agree that our study was not specifically designed to validate the diagnostic performance of the urine ELISA and FECT methods. The primary objective was to assess changes in infection prevalence, incidence, and reinfection following praziquantel treatment rather than to estimate diagnostic accuracy parameters. Therefore, we have removed the diagnostic accuracy section and instead mentioned the differences in diagnostic performance between the two methods in the Discussion section, drawing on evidence from previous studies.

*Fig 2: At least in the reviewer version of the manuscript, the background is all black. Was it supposed to be lines/arrows between the boxes?

**Response**:

We have revised Figure 2 to correct the background display problem and ensure that all figure elements, including boxes, connecting lines/arrows, and text, are clearly visible. The revised figure has been uploaded with the resubmitted manuscript.

Minor:
*Line 65; CCA should be written in full the first time i appears (it is written in full in line 68).

**Response**:

We have revised the manuscript to define cholangiocarcinoma (CCA) in full at its first occurrence in the text and have ensured that the abbreviation is used consistently thereafter. A correction was made in the introduction section, line 62.

*Line 427-429 (The outcome..) The sentence is difficult to understand, suggest rephrase.

**Response**:

We agree that the original sentence was unclear. We have revised the text for clarity as follows:

" The present study found that participants with a history of praziquantel treatment had a significantly higher likelihood of *O. viverrini* infection compared with those who had never received treatment. Individuals who reported one or more prior praziquantel treatments had a 1.49-fold higher risk of infection, consistent with findings from previous studies [43].

**Respond to the Reviewer #3 (highlighted in yellow)**

Reviewer #3: The manuscript entitled "Transmission dynamics and risk factor analysis of *Opisthorchis viverrini* infection after selective praziquantel treatment: evidence from a urine antigen assay and fecal examination in an endemic community in Northeast Thailand" reports the results of a longitudinal study assessing *O. viverrini* infection status using urine antigen ELISA and FECT in a rural endemic area of Thailand at baseline, 4 weeks post-treatment, and 24 weeks post-treatment. Overall, there are relatively few publications addressing this topic, and the study could contribute valuable information to the field. However, several major issues need to be addressed before the manuscript can be considered for publication.
Major comments

**Comments 1:** Use of the term "transmission dynamics"
The title and framing of the manuscript emphasize "transmission dynamics." However, with only baseline and 24-week follow-up measurements, the study design is not sufficient to characterize transmission dynamics in the strict epidemiologic sense. The data allow assessment of longitudinal change, cumulative incidence, reinfection, or infection acquisition over time, but not detailed transmission dynamics. The title and objectives should therefore be revised to better reflect the actual study design and analytical scope.

**Response 1:**

Thank you for pointing this out. We agree with this comment. Therefore, we have revised the title, objectives, and relevant sections of the manuscript to focus more appropriately on longitudinal changes in *O. viverrini* infection, reinfection, incidence, and associated risk factors following selective praziquantel treatment.

**Comments 2:** Unclear description of study population, sampling and grouping strategy
The Methods and Results sections do not clearly explain the study population and diagnostic group definitions. In particular, the definitions of the study groups appear inconsistent and overlapping. For example:
• Group 1 is described as "participants who were *O. viverrini* antigen negative and/or *O. viverrini* eggs positive"
• The other group is described as "participants who were *O. viverrini* antigen negative and/or *O. viverrini* eggs negative"
These definitions are confusing and potentially overlapping due to the use of "and/or." The grouping criteria should be clearly and consistently defined throughout the manuscript and figures.

**Response 2:**

Thank you for this important comment. We have revised the Methods (lines 128-137) and figures to clearly define the study groups and ensure that the grouping criteria are mutually exclusive and consistently applied throughout the manuscript.

Specifically, participants were classified by baseline infection status using the combined results of urine antigen ELISA and fecal examination. Group 1 comprised participants who tested positive for *O. viverrini* infection at baseline by **either** urine antigen ELISA **or** fecal examination and therefore subjected to praziquantel treatment. Group 2 comprised participants who tested negative by urine antigen ELISA and fecal examination at baseline and did not receive treatment. The revised definitions are now used consistently throughout the manuscript and accompanying figures.

In addition, Line111 states “A random household sample of the sub-district population aged 15 years or older was contacted and invited to participate”. However, the sampling methodology is insufficiently described. The authors should clearly specify the sampling frame (e.g., census list, household registry, village roster) and the sampling strategy used (e.g., simple random sampling, cluster sampling, multistage sampling, or systematic sampling) to allow readers to assess the representativeness of the study population and potential selection bias.

**Response:**

We have revised the Methods section to clarify the participant recruitment process and eligibility criteria in lines 109-113.

**Comments 3:** Inconsistency regarding reinfection period
The manuscript refers to reinfection occurring over 24 weeks; however, based on the study design, reinfection appears to have occurred during the 20-week interval between the 4-week post-treatment assessment and the 24-week follow-up. For example:
• Table 5 title states: "(combined incidence and/or reinfection) over a period of 24 weeks of the study"

• Similar wording appears in Line 374.
This should be corrected for accuracy. In general, the description of the study timeline and participant flow requires clearer presentation.

**Response 3:**

We have revised the manuscript to clarify the study timeline and participant flow. In particular, the title of Table 4 (Line 347) has been modified to more accurately describe the outcome as infection detected at the 24-week follow-up, and corresponding revisions have been made throughout the text to ensure consistency.

**Comments 4:** Statistical terminology not defined
The abbreviation "aRR" (adjusted relative risk) is used but not defined in the manuscript. Statistical terms and measures should be clearly introduced when first mentioned.

**Response 4:**

We have defined the abbreviation “aRR” (adjusted relative risk) at its first occurrence in the Methods section (Line 228).

**Comments 5:** Mismatch between stated objectives and presented analyses. The manuscript does not consistently focus on the stated aims of transmission dynamics and risk factor analysis. For example, Table 4 mainly presents diagnostic accuracy of urinary antigen ELISA compared with fecal examination at baseline and 24 weeks post-treatment. While potentially useful, this analysis does not directly address the stated primary objectives and appears disconnected from the main narrative. The authors should either better integrate these analyses into the study aims or reconsider the organization and focus of the manuscript.
Given the concerns above, particularly regarding conceptual framing, study description, and consistency of reporting, I do not currently recommend acceptance of this manuscript in its present form.

**Response 5:**

Thank you for this valuable comment. We agree that our study was not specifically designed to validate the diagnostic performance of the urine ELISA and FECT methods. The primary objective was to assess changes in infection prevalence, incidence, and reinfection following praziquantel treatment rather than to estimate diagnostic accuracy parameters. Therefore, we have revised the manuscript by removing the detailed diagnostic accuracy section and instead discussing the differences in diagnostic performance between the two methods in the Discussion section, drawing on evidence from previous studies.

Minor comments
1. Line 65: "CCA" should be written out in full at first mention.

**Response**

We have revised the manuscript to define cholangiocarcinoma (CCA) in full at its first occurrence in the text and have ensured that the abbreviation is used consistently thereafter. A correction was made in the introduction section, line 62.

2. Typographical error in Supplementary Figure S1: the word "infection" is misspelled.

**Response**

We have corrected the spelling of the word "infection" in Supplementary Figure S1 and carefully reviewed the figure for any additional typographical errors.

3. Line 220: "48 weeks post-treatment" is mentioned, but no such follow-up time point is described in Figure 2 or elsewhere.

**Response**

We agree that the reference to "48 weeks post-treatment" was incorrect. The text has been corrected to "24 weeks" to align with the study design, Figure 2, and the follow-up schedule described throughout the manuscript.

4. Line 263: "Among the antigen-positive participants (group 1, n = 244)" is inconsistent with Figure 2, where Group 1 appears to include participants who were antigen-positive and/or stool-positive.

**Response**

The text is revised to accurately reflect the definition of Group 1 as participants who tested positive for *O. viverrini* infection at baseline by either urine antigen ELISA or fecal examination. Consistent terminology is now used throughout the manuscript and figures.

5. Figures 3, 4, and 5 are of poor resolution and should be improved for publication quality.

**Response**

We have replaced these figures with higher-resolution versions and checked that all text, symbols, axes, and graphical elements are clearly visible and suitable for publication quality in the revised manuscript.
