# Supplementary material for: Longitudinal changes and risk factors of Opisthorchis viverrini infection after selective praziquantel treatment: evidence from urine antigen assay and fecal examination in an endemic community in Northeast Thailand
Source: PLoS One. 2026 Jul 6;21(7):e0352854. doi: 10.1371/journal.pone.0352854 (PMC13336184; doi:10.1371/journal.pone.0352854)
Supplement: Peer review history

# Peer review: Decision Letter 0

**DOI:** 10.1371/journal.pone.0352854.r001

**Authors:** Petr Heneberg

25 May 2026

PONE-D-26-20582

Transmission dynamics and risk factor analysis of Opisthorchis viverrini infection after selective praziquantel treatment: evidence from a urine antigen assay and fecal examination in an endemic community in Northeast Thailand

PLOS One

Dear Dr. Kopolrat,

Thank you for submitting your manuscript to PLOS ONE. After careful consideration, we feel that it has merit but does not fully meet PLOS ONE’s publication criteria as it currently stands. Therefore, we invite you to submit a revised version of the manuscript that addresses the points raised during the review process.

Please submit your revised manuscript by Jul 09 2026 11:59PM. If you will need more time than this to complete your revisions, please reply to this message or contact the journal office at plosone@plos.org. When you're ready to submit your revision, log on to https://www.editorialmanager.com/pone/ and select the 'Submissions Needing Revision' folder to locate your manuscript file.

Please include the following items when submitting your revised manuscript:

- A letter that responds to each point raised by the academic editor and reviewer(s). You should upload this letter as a separate file labeled 'Response to Reviewers'.
- A marked-up copy of your manuscript that highlights changes made to the original version. You should upload this as a separate file labeled 'Revised Manuscript with Track Changes'.
- An unmarked version of your revised paper without tracked changes. You should upload this as a separate file labeled 'Manuscript'.

If you would like to make changes to your financial disclosure, please include your updated statement in your cover letter. Guidelines for resubmitting your figure files are available below the reviewer comments at the end of this letter.

If applicable, we recommend that you deposit your laboratory protocols in protocols.io to enhance the reproducibility of your results. Protocols.io assigns your protocol its own identifier (DOI) so that it can be cited independently in the future. For instructions see: https://journals.plos.org/plosone/s/submission-guidelines#loc-laboratory-protocols. Additionally, PLOS ONE offers an option for publishing peer-reviewed Lab Protocol articles, which describe protocols hosted on protocols.io. Read more information on sharing protocols at https://plos.org/protocols?utm_medium=editorial-email&utm_source=authorletters&utm_campaign=protocols.

As the corresponding author, your ORCID iD is verified in the submission system and will appear in the published article. PLOS supports the use of ORCID, and we encourage all coauthors to register for an ORCID iD and use it as well. Please encourage your coauthors to verify their ORCID iD within the submission system before final acceptance, as unverified ORCID iDs will not appear in the published article. *Only*  the individual author can complete the verification step; PLOS staff *cannot*  verify ORCID iDs on behalf of authors.

We look forward to receiving your revised manuscript.

Kind regards,

Petr Heneberg

Academic Editor

PLOS One

Journal Requirements:

When submitting your revision, we need you to address these additional requirements.

1. Please ensure that your manuscript meets PLOS ONE's style requirements, including those for file naming. The PLOS ONE style templates can be found at

https://journals.plos.org/plosone/s/file?id=wjVg/PLOSOne_formatting_sample_main_body.pdf and

https://journals.plos.org/plosone/s/file?id=ba62/PLOSOne_formatting_sample_title_authors_affiliations.pdf.

2. Thank you for stating the following financial disclosure:

“The Kasetsart University Research and Development Institute, KURDI (Grant number YF(KU)53.66), and Fluke-Free Thailand, National Research Council of Thailand. “

Please state what role the funders took in the study.  If the funders had no role, please state: "The funders had no role in study design, data collection and analysis, decision to publish, or preparation of the manuscript."

If this statement is not correct you must amend it as needed.

Please include this amended Role of Funder statement in your cover letter; we will change the online submission form on your behalf.

3. In this instance it seems there may be acceptable restrictions in place that prevent the public sharing of your minimal data. However, in line with our goal of ensuring long-term data availability to all interested researchers, PLOS’ Data Policy states that authors cannot be the sole named individuals responsible for ensuring data access (http://journals.plos.org/plosone/s/data-availability#loc-acceptable-data-sharing-methods).

Data requests to a non-author institutional point of contact, such as a data access or ethics committee, helps guarantee long term stability and availability of data. Providing interested researchers with a durable point of contact ensures data will be accessible even if an author changes email addresses, institutions, or becomes unavailable to answer requests.

Before we proceed with your manuscript, please also provide non-author contact information (phone/email/hyperlink) for a data access committee, ethics committee, or other institutional body to which data requests may be sent. If no institutional body is available to respond to requests for your minimal data, please consider if there any institutional representatives who did not collaborate in the study, and are not listed as authors on the manuscript, who would be able to hold the data and respond to external requests for data access? If so, please provide their contact information (i.e., email address). Please also provide details on how you will ensure persistent or long-term data storage and availability.

4. Please be informed that funding information should not appear in the Acknowledgments section or other areas of your manuscript. We will only publish funding information present in the Funding Statement section of the online submission form. Please remove any funding-related text from the manuscript.

5. We note that Figure 1 in your submission contain [map/satellite] images which may be copyrighted. All PLOS content is published under the Creative Commons Attribution License (CC BY 4.0), which means that the manuscript, images, and Supporting Information files will be freely available online, and any third party is permitted to access, download, copy, distribute, and use these materials in any way, even commercially, with proper attribution. For these reasons, we cannot publish previously copyrighted maps or satellite images created using proprietary data, such as Google software (Google Maps, Street View, and Earth). For more information, see our copyright guidelines: http://journals.plos.org/plosone/s/licenses-and-copyright.

We require you to either (1) present written permission from the copyright holder to publish these figures specifically under the CC BY 4.0 license, or (2) remove the figures from your submission:

a. You may seek permission from the original copyright holder of Figure 1 to publish the content specifically under the CC BY 4.0 license.

We recommend that you contact the original copyright holder with the Content Permission Form (http://journals.plos.org/plosone/s/file?id=7c09/content-permission-form.pdf) and the following text:

“I request permission for the open-access journal PLOS ONE to publish XXX under the Creative Commons Attribution License (CCAL) CC BY 4.0 (http://creativecommons.org/licenses/by/4.0/). Please be aware that this license allows unrestricted use and distribution, even commercially, by third parties. Please reply and provide explicit written permission to publish XXX under a CC BY license and complete the attached form.”

Please upload the completed Content Permission Form or other proof of granted permissions as an "Other" file with your submission.

In the figure caption of the copyrighted figure, please include the following text: “Reprinted from [ref] under a CC BY license, with permission from [name of publisher], original copyright [original copyright year].”

b. If you are unable to obtain permission from the original copyright holder to publish these figures under the CC BY 4.0 license or if the copyright holder’s requirements are incompatible with the CC BY 4.0 license, please either i) remove the figure or ii) supply a replacement figure that complies with the CC BY 4.0 license. Please check copyright information on all replacement figures and update the figure caption with source information. If applicable, please specify in the figure caption text when a figure is similar but not identical to the original image and is therefore for illustrative purposes only.

The following resources for replacing copyrighted map figures may be helpful:

USGS National Map Viewer (public domain): http://viewer.nationalmap.gov/viewer/

The Gateway to Astronaut Photography of Earth (public domain): http://eol.jsc.nasa.gov/sseop/clickmap/

Maps at the CIA (public domain): https://www.cia.gov/library/publications/the-world-factbook/index.html and https://www.cia.gov/library/publications/cia-maps-publications/index.html

NASA Earth Observatory (public domain): http://earthobservatory.nasa.gov/

Landsat: http://landsat.visibleearth.nasa.gov/

USGS EROS (Earth Resources Observatory and Science (EROS) Center) (public domain): http://eros.usgs.gov/#

Natural Earth (public domain): http://www.naturalearthdata.com/

6. If the reviewer comments include a recommendation to cite specific previously published works, please review and evaluate these publications to determine whether they are relevant and should be cited. There is no requirement to cite these works unless the editor has indicated otherwise.

[Note: HTML markup is below. Please do not edit.]

Reviewers' comments:

Reviewer's Responses to Questions

**Comments to the Author**

1. Is the manuscript technically sound, and do the data support the conclusions?

The manuscript must describe a technically sound piece of scientific research with data that supports the conclusions. Experiments must have been conducted rigorously, with appropriate controls, replication, and sample sizes. The conclusions must be drawn appropriately based on the data presented.

Reviewer #1: Yes

Reviewer #2: Partly

Reviewer #3: Partly

**********

2. Has the statistical analysis been performed appropriately and rigorously?

Reviewer #1: Yes

Reviewer #2: Yes

Reviewer #3: No

**********

3. Have the authors made all data underlying the findings in their manuscript fully available?

The PLOS Data policy requires authors to make all data underlying the findings described in their manuscript fully available without restriction, with rare exception (please refer to the Data Availability Statement in the manuscript PDF file). The data should be provided as part of the manuscript or its supporting information, or deposited to a public repository. For example, in addition to summary statistics, the data points behind means, medians and variance measures should be available. If there are restrictions on publicly sharing data—e.g. participant privacy or use of data from a third party—those must be specified.

Reviewer #1: Yes

Reviewer #2: No

Reviewer #3: No

**********

4. Is the manuscript presented in an intelligible fashion and written in standard English?

PLOS ONE does not copyedit accepted manuscripts, so the language in submitted articles must be clear, correct, and unambiguous. Any typographical or grammatical errors should be corrected at revision, so please note any specific errors here.

Reviewer #1: Yes

Reviewer #2: Yes

Reviewer #3: Yes

**********

5. Review Comments to the Author

Please use the space provided to explain your answers to the questions above. You may also include additional comments for the author, including concerns about dual publication, research ethics, or publication ethics. (Please upload your review as an attachment if it exceeds 20,000 characters)

Reviewer #1: The manuscript presents a strong and well-designed epidemiological study investigating the transmission dynamics of Opisthorchis viverrini infection in an endemic region of Northeast Thailand. The prospective cohort design, the analysis of both incidence and reinfection, and the integration of urine antigen detection together provide important and highly interesting findings with clear public health relevance. In particular, the application of urinary antigen ELISA for monitoring transmission dynamics represents an important contribution to the field and highlights the potential advantages of non-invasive diagnostic approaches in opisthorchiasis surveillance. The manuscript is generally well written, scientifically sound, and supported by robust epidemiological data. The discussion is balanced and appropriately cautious. However, I would ask the authors to further discuss several important methodological and interpretational issues before publication.

1. FECT as an imperfect reference standard. This is probably the most important methodological issue of the manuscript. The diagnostic performance of the urine ELISA is evaluated against FECT, although FECT itself has relatively low sensitivity and cannot reliably distinguish O. viverrini eggs from minute intestinal flukes. Therefore, sensitivity, specificity, kappa values, and false-positive calculations may be distorted by the limitations of the reference standard itself. The authors partially acknowledge this issue; however, I recommend emphasizing this point more clearly in the Discussion section. In particular, the following statement or a similar interpretation should be considered: “Because FECT is an imperfect reference standard, the reported diagnostic performance metrics of urine ELISA should be interpreted cautiously.”

2. Interpretation of ELISA-positive cases: The manuscript would benefit from a more detailed discussion regarding the biological interpretation of urine antigen positivity. It is not entirely certain that every ELISA-positive individual represents an active egg-producing infection. Urinary antigen positivity may also reflect residual circulating antigens, delayed antigen clearance after treatment, or possibly very early-stage infection before detectable egg shedding. This important immunological and diagnostic aspect deserves further discussion.

3. Reinfection versus treatment failure: The manuscript interprets positivity at 24 weeks mainly as reinfection. However, it remains unclear whether all cases truly represent reinfection or whether some individuals may have experienced partial treatment failure, persistent low-level infection, or subcurative praziquantel response. I recommend that the authors discuss this distinction more thoroughly, as it may influence the interpretation of reinfection dynamics.

4. Increased risk associated with previous praziquantel treatment: The finding that prior praziquantel treatment is associated with higher infection risk is epidemiologically very interesting. However, this association may be strongly confounded. It is possible that individuals with repeated praziquantel treatments are simply those with the highest long-term exposure risk and most persistent behavioural risk factors. Therefore, previous praziquantel treatment history may function more as a marker of repeated exposure rather than an independent biological risk factor. This interpretation should be further elaborated in the Discussion.

5. Duration of follow-up and potential seasonal effects: The 6-month follow-up period is appropriate for evaluating short-term epidemiological dynamics; however, it may not fully capture seasonal transmission patterns. I would ask the authors to comment on whether the study period coincided with specific environmental or sociocultural factors in the region, such as: fishing seasons, rainy season-related ecological changes, national or local festivals involving traditional raw fish consumption. These factors could potentially influence transmission intensity and reinfection rates and would strengthen the epidemiological interpretation of the findings.

Overall, this is an important and valuable study with strong epidemiological content and highly relevant findings. After addressing the points above, I believe the manuscript would make a meaningful contribution to the field.

Reviewer #2: Dear authors. I've read this manuscript with interest. Congratulations. I was surprised to see that during the 24 w study period, the incidense was higher in those negative at baseline (hence not infected during the previous years) than the reinfection rate in those positive at baseline. But that is what the numbers show.

The manuscript is well written in fluent language.

Major:

*The main problem that I find is the relation between the two methods; urinary ELISA and FECT. Using the ELISA it is a 5-10-fold (!) increase in prevalence/incidense/reinfection rate. It is well known (as written in the manuscript) that FECT is an imperfect 'gold standard' with both false positives and especially false negatives. It is almost comparing apples and oranges and make the section The diagnostic accuracy difficult (and unnecessary??). In line 324 it states ELISA has sensitivity 61.9%, which is not so good when the number of positive ELISA is so much higher than FECT (due to false positive 'gold standard'/FECT?).

Table 4 mix just one technical test quantification (sensitivity) and one prevalencedependent diagnostic predictive value (NPV).

In line 328 it says: 'The positive urine ELISA results among fecal egg-negative individuals (i.e. false positives) ranged from 0.1% to 1.6%.' How do you calculate those numbers? There is a 5-10-fold higher number of of ELISA positives than FECT positives.

I suggest the diagnostic accuracy section is rewritten for clarity. As the study is not really designed to validate the test properties of the two diagnostic methods, you may alternativly consider if the section can be omitted and the difference in test properties be discussed in Discussion, based on findings from previous studies. Especially interesting is the assumed specificity of ELISA (many false positives?)

*Fig 2: At least in the reviewer version of the manuscript the background is all black. Was it supposed to be lines/arrows between the boxes?

Minor:

*Line 65; CCA should be written in full the first time i appears ( it is written in full in line 68).

*Line 427-429 (The outcome..) The sentence is difficult to understand, suggest rephrase.

Reviewer #3: The manuscript entitled "Transmission dynamics and risk factor analysis of Opisthorchis viverrini infection after selective praziquantel treatment: evidence from a urine antigen assay and fecal examination in an endemic community in Northeast Thailand" reports the results of a longitudinal study assessing O. viverrini infection status using urine antigen ELISA and FECT in a rural endemic area of Thailand at baseline, 4 weeks post-treatment, and 24 weeks post-treatment. Overall, there are relatively few publications addressing this topic, and the study could contribute valuable information to the field. However, several major issues need to be addressed before the manuscript can be considered for publication.

Major comments

1. Use of the term "transmission dynamics"

The title and framing of the manuscript emphasize "transmission dynamics." However, with only baseline and 24-week follow-up measurements, the study design is not sufficient to characterize transmission dynamics in the strict epidemiologic sense. The data allow assessment of longitudinal change, cumulative incidence, reinfection, or infection acquisition over time, but not detailed transmission dynamics. The title and objectives should therefore be revised to better reflect the actual study design and analytical scope.

2. Unclear description of study population, sampling and grouping strategy

The Methods and Results sections do not clearly explain the study population and diagnostic group definitions. In particular, the definitions of the study groups appear inconsistent and overlapping. For example:

• Group 1 is described as "participants who were O. viverrini antigen negative and/or O. viverrini eggs positive"

• The other group is described as "participants who were O. viverrini antigen negative and/or O. viverrini eggs negative"

These definitions are confusing and potentially overlapping due to the use of "and/or." The grouping criteria should be clearly and consistently defined throughout the manuscript and figures.

In addition, Line111 states “A random household sample of the sub-district population aged 15 years or older was contacted and invited to participate”. However, the sampling methodology is insufficiently described. The authors should clearly specify the sampling frame (e.g., census list, household registry, village roster) and the sampling strategy used (e.g., simple random sampling, cluster sampling, multistage sampling, or systematic sampling) to allow readers to assess the representativeness of the study population and potential selection bias.

3. Inconsistency regarding reinfection period

The manuscript refers to reinfection occurring over 24 weeks; however, based on the study design, reinfection appears to have occurred during the 20-week interval between the 4-week post-treatment assessment and the 24-week follow-up. For example:

• Table 5 title states: "(combined incidence and/or reinfection) over a period of 24 weeks of the study"

• Similar wording appears in Line 374.

This should be corrected for accuracy. In general, the description of the study timeline and participant flow requires clearer presentation.

4. Statistical terminology not defined

The abbreviation "aRR" (adjusted relative risk) is used but not defined in the manuscript. Statistical terms and measures should be clearly introduced when first mentioned.

5. Mismatch between stated objectives and presented analyses

The manuscript does not consistently focus on the stated aims of transmission dynamics and risk factor analysis. For example, Table 4 mainly presents diagnostic accuracy of urinary antigen ELISA compared with fecal examination at baseline and 24 weeks post-treatment. While potentially useful, this analysis does not directly address the stated primary objectives and appears disconnected from the main narrative. The authors should either better integrate these analyses into the study aims or reconsider the organization and focus of the manuscript.

Given the concerns above, particularly regarding conceptual framing, study description, and consistency of reporting, I do not currently recommend acceptance of this manuscript in its present form.

Minor comments

1. Line 65: "CCA" should be written out in full at first mention.

2. Typographical error in Supplementary Figure S1: the word "infection" is misspelled.

3. Line 220: "48 weeks post-treatment" is mentioned, but no such follow-up time point is described in Figure 2 or elsewhere.

4. Line 263: "Among the antigen-positive participants (group 1, n = 244)" is inconsistent with Figure 2, where Group 1 appears to include participants who were antigen-positive and/or stool-positive.

5. Figures 3, 4, and 5 are of poor resolution and should be improved for publication quality.

**********

6. PLOS authors have the option to publish the peer review history of their article (what does this mean?). If published, this will include your full peer review and any attached files.

If you choose “no”, your identity will remain anonymous but your review may still be made public.

**Do you want your identity to be public for this peer review?**  For information about this choice, including consent withdrawal, please see our Privacy Policy.

Reviewer #1: No

Reviewer #2: No

Reviewer #3: No

**********

[NOTE: If reviewer comments were submitted as an attachment file, they will be attached to this email and accessible via the submission site. Please log into your account, locate the manuscript record, and check for the action link "View Attachments". If this link does not appear, there are no attachment files.]

To ensure your figures meet our technical requirements, please review our figure guidelines: https://journals.plos.org/plosone/s/figures

You may also use PLOS’s free figure tool, NAAS, to help you prepare publication quality figures: https://journals.plos.org/plosone/s/figures#loc-tools-for-figure-preparation.

NAAS will assess whether your figures meet our technical requirements by comparing each figure against our figure specifications.

# Author comment: Author response to Decision Letter 1

**DOI:** 10.1371/journal.pone.0352854.r002

8 Jun 2026

#1 Respond to the Editor

1. Please ensure that your manuscript meets PLOS ONE's style requirements, including those for file naming. The PLOS ONE style templates can be found at

https://journals.plos.org/plosone/s/file?id=wjVg/PLOSOne_formatting_sample_main_body.pdf and

https://journals.plos.org/plosone/s/file?id=ba62/PLOSOne_formatting_sample_title_authors_affiliations.pdf.

Response 1:

Thank you for bringing this to our attention. We have carefully reviewed the manuscript and revised it to comply with PLOS ONE formatting and style requirements, including manuscript structure, section headings, references, tables, figures, and file-naming conventions, in accordance with the journal's guidelines and templates.

2. Thank you for stating the following financial disclosure:

“The Kasetsart University Research and Development Institute, KURDI (Grant number YF(KU)53.66), and Fluke-Free Thailand, National Research Council of Thailand. “

Please state what role the funders took in the study. If the funders had no role, please state: "The funders had no role in study design, data collection and analysis, decision to publish, or preparation of the manuscript."

If this statement is not correct you must amend it as needed.

Please include this amended Role of Funder statement in your cover letter; we will change the online submission form on your behalf.

Response 2:

Thank you for this comment. We confirm that the funders had no role in study design, data collection and analysis, decision to publish, or preparation of the manuscript. We have revised the Financial Disclosure statement accordingly.

3. In this instance it seems there may be acceptable restrictions in place that prevent the public sharing of your minimal data. However, in line with our goal of ensuring long-term data availability to all interested researchers, PLOS’ Data Policy states that authors cannot be the sole named individuals responsible for ensuring data access (http://journals.plos.org/plosone/s/data-availability#loc-acceptable-data-sharing-methods).

Data requests to a non-author institutional point of contact, such as a data access or ethics committee, helps guarantee long term stability and availability of data. Providing interested researchers with a durable point of contact ensures data will be accessible even if an author changes email addresses, institutions, or becomes unavailable to answer requests.

Before we proceed with your manuscript, please also provide non-author contact information (phone/email/hyperlink) for a data access committee, ethics committee, or other institutional body to which data requests may be sent. If no institutional body is available to respond to requests for your minimal data, please consider if there any institutional representatives who did not collaborate in the study, and are not listed as authors on the manuscript, who would be able to hold the data and respond to external requests for data access? If so, please provide their contact information (i.e., email address). Please also provide details on how you will ensure persistent or long-term data storage and availability.

Response 3:

Thank you for this comment. All relevant data underlying the findings of this study are provided within the manuscript and its Supporting Information files.

4. Please be informed that funding information should not appear in the Acknowledgments section or other areas of your manuscript. We will only publish funding information present in the Funding Statement section of the online submission form. Please remove any funding-related text from the manuscript.

Response 4:

We have reviewed the manuscript to ensure compliance with PLOS ONE's funding disclosure requirements. The Acknowledgments section contains only acknowledgments of study participants and collaborating institutions and does not include any funding-related information. Funding details are reported exclusively in the Funding Statement section of the online submission form.

5. We note that Figure 1 in your submission contain [map/satellite] images which may be copyrighted. All PLOS content is published under the Creative Commons Attribution License (CC BY 4.0), which means that the manuscript, images, and Supporting Information files will be freely available online, and any third party is permitted to access, download, copy, distribute, and use these materials in any way, even commercially, with proper attribution. For these reasons, we cannot publish previously copyrighted maps or satellite images created using proprietary data, such as Google software (Google Maps, Street View, and Earth). For more information, see our copyright guidelines: http://journals.plos.org/plosone/s/licenses-and-copyright.

We require you to either (1) present written permission from the copyright holder to publish these figures specifically under the CC BY 4.0 license, or (2) remove the figures from your submission:

a. You may seek permission from the original copyright holder of Figure 1 to publish the content specifically under the CC BY 4.0 license.

We recommend that you contact the original copyright holder with the Content Permission Form (http://journals.plos.org/plosone/s/file?id=7c09/content-permission-form.pdf) and the following text:

“I request permission for the open-access journal PLOS ONE to publish XXX under the Creative Commons Attribution License (CCAL) CC BY 4.0 (http://creativecommons.org/licenses/by/4.0/). Please be aware that this license allows unrestricted use and distribution, even commercially, by third parties. Please reply and provide explicit written permission to publish XXX under a CC BY license and complete the attached form.”

Please upload the completed Content Permission Form or other proof of granted permissions as an "Other" file with your submission.

In the figure caption of the copyrighted figure, please include the following text: “Reprinted from [ref] under a CC BY license, with permission from [name of publisher], original copyright [original copyright year].”

b. If you are unable to obtain permission from the original copyright holder to publish these figures under the CC BY 4.0 license or if the copyright holder’s requirements are incompatible with the CC BY 4.0 license, please either i) remove the figure or ii) supply a replacement figure that complies with the CC BY 4.0 license. Please check copyright information on all replacement figures and update the figure caption with source information. If applicable, please specify in the figure caption text when a figure is similar but not identical to the original image and is therefore for illustrative purposes only.

The following resources for replacing copyrighted map figures may be helpful:

USGS National Map Viewer (public domain): http://viewer.nationalmap.gov/viewer/

The Gateway to Astronaut Photography of Earth (public domain): http://eol.jsc.nasa.gov/sseop/clickmap/

Maps at the CIA (public domain): https://www.cia.gov/library/publications/the-world-factbook/index.html and https://www.cia.gov/library/publications/cia-maps-publications/index.html

NASA Earth Observatory (public domain): http://earthobservatory.nasa.gov/

Landsat: http://landsat.visibleearth.nasa.gov/

USGS EROS (Earth Resources Observatory and Science (EROS) Center) (public domain): http://eros.usgs.gov/#

Natural Earth (public domain): http://www.naturalearthdata.com/

Response 5:

Thank you for this comment. We have reviewed Figure 1 to ensure compliance with the PLOS ONE copyright and licensing requirements. Figure 1 was created by the authors using QGIS software, which is distributed under the Creative Commons Attribution 4.0 International License. No copyrighted Google Maps, Google Earth, or other proprietary map or satellite imagery was used in the preparation of this figure. We have revised the manuscript to clarify the source of the map and confirm that the figure complies with the CC BY 4.0 licensing requirements.

6. If the reviewer comments include a recommendation to cite specific previously published works, please review and evaluate these publications to determine whether they are relevant and should be cited. There is no requirement to cite these works unless the editor has indicated otherwise.

Response 6:

Thank you for this guidance. We confirm that we have reviewed all reviewer comments and note that no specific additional references were requested. We have ensured that the manuscript cites the most relevant literature to support the study background, methods, and interpretation of findings.

Respond to the Reviewer #1 (highlighted in blue)

Reviewer #1: The manuscript presents a strong and well-designed epidemiological study investigating the transmission dynamics of Opisthorchis viverrini infection in an endemic region of Northeast Thailand. The prospective cohort design, the analysis of both incidence and reinfection, and the integration of urine antigen detection together provide important and highly interesting findings with clear public health relevance. In particular, the application of urinary antigen ELISA for monitoring transmission dynamics represents an important contribution to the field and highlights the potential advantages of non-invasive diagnostic approaches in opisthorchiasis surveillance. The manuscript is generally well written, scientifically sound, and supported by robust epidemiological data. The discussion is balanced and appropriately cautious. However, I would ask the authors to further discuss several important methodological and interpretational issues before publication.

Comments 1: FECT as an imperfect reference standard. This is probably the most important methodological issue of the manuscript. The diagnostic performance of the urine ELISA is evaluated against FECT, although FECT itself has relatively low sensitivity and cannot reliably distinguish O. viverrini eggs from minute intestinal flukes. Therefore, sensitivity, specificity, kappa values, and false-positive calculations may be distorted by the limitations of the reference standard itself. The authors partially acknowledge this issue; however, I recommend emphasizing this point more clearly in the Discussion section. In particular, the following statement or a similar interpretation should be considered: “Because FECT is an imperfect reference standard, the reported diagnostic performance metrics of urine ELISA should be interpreted cautiously.”

Response 1:

Thank you for this helpful suggestion. Accordingly, we have revised the Discussion section to explicitly state that FECT is an imperfect reference standard and may fail to detect low-intensity infections, and hence the reported diagnostic performance measures of the urine ELISA should therefore be interpreted with caution (Discussion section, lines 407-410).

Comments 2: Interpretation of ELISA-positive cases: The manuscript would benefit from a more detailed discussion regarding the biological interpretation of urine antigen positivity. It is not entirely certain that every ELISA-positive individual represents an active egg-producing infection. Urinary antigen positivity may also reflect residual circulating antigens, delayed antigen clearance after treatment, or possibly very early-stage infection before detectable egg shedding. This important immunological and diagnostic aspect deserves further discussion.

Response 2:

Thank you for this insightful comment in adding more reasons and interpretations of urine antigen positivity. We have revised the Discussion section to acknowledge that a positive urine ELISA result may not entirely indicate an active egg-producing infection and may reflect residual circulating antigens, delayed antigen clearance following treatment, or early-stage infection prior to detectable egg shedding. We have also noted that these situations may contribute to discordance between urine antigen detection and fecal examination in the longitudinal study. (Discussion section, lines 410-418).

Comments 3: Reinfection versus treatment failure: The manuscript interprets positivity at 24 weeks mainly as reinfection. However, it remains unclear whether all cases truly represent reinfection or whether some individuals may have experienced partial treatment failure, persistent low-level infection, or subcurative praziquantel response. I recommend that the authors discuss this distinction more thoroughly, as it may influence the interpretation of reinfection dynamics.

Response 3:

Thank you for this important comment. In our study, reinfection was assessed only among the 220 participants who were antigen- or egg-negative at 4 weeks post-treatment with praziquantel. Thus, the reinfection rate was calculated among individuals considered cured according to the study's diagnostic criteria. Indeed, 166 of 220 individuals were consistently antigen-negative at both the 4- and 24-week follow-ups, suggesting true curative treatment. Nevertheless, we acknowledge that a negative result at week 4 does not completely exclude the possibility of residual low-intensity infection below the detection limit of the diagnostic methods used. Therefore, some positive cases detected at week 24 may have represented persistent low-level infection, partial treatment failure, or subcurative response to praziquantel rather than true reinfection alone. Because no additional diagnostic assessments were conducted between weeks 4 and 24, these possibilities cannot be completely ruled out. We have revised the Discussion section to acknowledge this limitation and to interpret reinfection estimates with appropriate caution (Lines 416-419).

Comments 4: Increased risk associated with previous praziquantel treatment: The finding that prior praziquantel treatment is associated with higher infection risk is epidemiologically very interesting. However, this association may be strongly confounded. It is possible that individuals with repeated praziquantel treatments are simply those with the highest long-term exposure risk and most persistent behavioural risk factors. Therefore, previous praziquantel treatment history may function more as a marker of repeated exposure rather than an independent biological risk factor. This interpretation should be further elaborated in the Discussion.

Response 4:

Thank you for this insightful comment. We agree that the observed association between prior praziquantel treatment and O. viverrini infection suggests potential reinfection especially in individuals with a history of repeated praziquantel treatment due to persistent exposure to infection due to continued consumption of raw fish. Therefore, a history of previous praziquantel treatment may serve as a marker of repeated exposure and reinfection risk rather than an independent biological risk factor. We have revised the Discussion section to clarify this interpretation and to acknowledge the potential for confounding by unmeasured behavioral and environmental factors (Lines 426-432).

Comments 5: Duration of follow-up and potential seasonal effects: The 6-month follow-up period is appropriate for evaluating short-term epidemiological dynamics; however, it may not fully capture seasonal transmission patterns. I would ask the authors to comment on whether the study period coincided with specific environmental or sociocultural factors in the region, such as fishing seasons, rainy season-related ecological changes, national or local festivals involving traditional raw fish consumption. These factors could potentially influence transmission intensity and reinfection rates and would strengthen the epidemiological interpretation of the findings.

Overall, this is an important and valuable study with strong epidemiological content and highly relevant findings. After addressing the points above, I believe the manuscript would make a meaningful contribution to the field.

Response 5:

Thank you for this valuable suggestion. We agree that seasonal and sociocultural factors may influence O. viverrini transmission and reinfection patterns. The study was conducted from April to November 2024, covering much of the rainy season in Northeastern Thailand (May-October), when freshwater ecosystems are most productive, and fishing activities are common. This period may increase access to and consumption

Attachment Submitted filename: Response to reviewer_R1.docx

# Editor report: Decision Letter 1

**DOI:** 10.1371/journal.pone.0352854.r003

**Authors:** Petr Heneberg, Petr Heneberg

10 Jun 2026

PONE-D-26-20582R1

Longitudinal changes and risk factors of Opisthorchis viverrini infection after selective praziquantel treatment: evidence from urine antigen assay and fecal examination in an endemic community in Northeast Thailand

PLOS One

Dear Dr. Kopolrat,

Thank you for submitting your manuscript to PLOS ONE. After careful consideration, we feel that it has merit but does not fully meet PLOS ONE’s publication criteria as it currently stands. Therefore, we invite you to submit a revised version of the manuscript that addresses the points raised during the review process.

Please submit your revised manuscript by Jul 25 2026 11:59PM. If you will need more time than this to complete your revisions, please reply to this message or contact the journal office at plosone@plos.org. When you're ready to submit your revision, log on to https://www.editorialmanager.com/pone/ and select the 'Submissions Needing Revision' folder to locate your manuscript file.

Please include the following items when submitting your revised manuscript:

- A letter that responds to each point raised by the academic editor and reviewer(s). You should upload this letter as a separate file labeled 'Response to Reviewers'.
- A marked-up copy of your manuscript that highlights changes made to the original version. You should upload this as a separate file labeled 'Revised Manuscript with Track Changes'.
- An unmarked version of your revised paper without tracked changes. You should upload this as a separate file labeled 'Manuscript'.

If you would like to make changes to your financial disclosure, please include your updated statement in your cover letter. Guidelines for resubmitting your figure files are available below the reviewer comments at the end of this letter.

If applicable, we recommend that you deposit your laboratory protocols in protocols.io to enhance the reproducibility of your results. Protocols.io assigns your protocol its own identifier (DOI) so that it can be cited independently in the future. For instructions see: https://journals.plos.org/plosone/s/submission-guidelines#loc-laboratory-protocols. Additionally, PLOS ONE offers an option for publishing peer-reviewed Lab Protocol articles, which describe protocols hosted on protocols.io. Read more information on sharing protocols at https://plos.org/protocols?utm_medium=editorial-email&utm_source=authorletters&utm_campaign=protocols.

As the corresponding author, your ORCID iD is verified in the submission system and will appear in the published article. PLOS supports the use of ORCID, and we encourage all coauthors to register for an ORCID iD and use it as well. Please encourage your coauthors to verify their ORCID iD within the submission system before final acceptance, as unverified ORCID iDs will not appear in the published article. *Only*  the individual author can complete the verification step; PLOS staff *cannot*  verify ORCID iDs on behalf of authors.

We look forward to receiving your revised manuscript.

Kind regards,

Petr Heneberg

Academic Editor

PLOS One

Journal Requirements:

If the reviewer comments include a recommendation to cite specific previously published works, please review and evaluate these publications to determine whether they are relevant and should be cited. There is no requirement to cite these works unless the editor has indicated otherwise.

Please review your reference list to ensure that it is complete and correct. If you have cited papers that have been retracted, please include the rationale for doing so in the manuscript text, or remove these references and replace them with relevant current references. Any changes to the reference list should be mentioned in the rebuttal letter that accompanies your revised manuscript. If you need to cite a retracted article, indicate the article’s retracted status in the References list and also include a citation and full reference for the retraction notice.

Additional Editor Comments:

Before we proceed, please address the following two points:

First, please verify the source and license of the underlying geospatial data used in Fig. 1. The response indicates that the figure was created in QGIS, but QGIS is the software used to prepare the map and does not by itself establish that the underlying map layers, boundaries, or other geospatial data are compatible with publication under the CC BY 4.0 license. Please add the actual data source(s) for the map layers to the figure caption and confirm that these data can be redistributed under the journal’s license.

Second, please harmonize the ethics and consent wording between the manuscript and the submission form. The submission ethics statement indicates that only participants who provided written informed consent were included, whereas the manuscript states that adults provided written informed consent and that parents or legal guardians provided verbal consent for participants aged 15–17 years. Please ensure that the wording is consistent and that the reported form of consent for minors matches the ethics approval.

Once these technical issues are corrected, no further scientific revision appears necessary.

[Note: HTML markup is below. Please do not edit.]

[NOTE: If reviewer comments were submitted as an attachment file, they will be attached to this email and accessible via the submission site. Please log into your account, locate the manuscript record, and check for the action link "View Attachments". If this link does not appear, there are no attachment files.]

To ensure your figures meet our technical requirements, please review our figure guidelines: https://journals.plos.org/plosone/s/figures

You may also use PLOS’s free figure tool, NAAS, to help you prepare publication quality figures: https://journals.plos.org/plosone/s/figures#loc-tools-for-figure-preparation.

NAAS will assess whether your figures meet our technical requirements by comparing each figure against our figure specifications.

# Author comment: Author response to Decision Letter 2

**DOI:** 10.1371/journal.pone.0352854.r004

15 Jun 2026

Journal Requirements:

If applicable, we recommend that you deposit your laboratory protocols in protocols.io to enhance the reproducibility of your results. Protocols.io assigns your protocol its own identifier (DOI) so that it can be cited independently in the future. For instructions see: https://journals.plos.org/plosone/s/submission-guidelines#loc-laboratory-protocols. Additionally, PLOS ONE offers an option for publishing peer-reviewed Lab Protocol articles, which describe protocols hosted on protocols.io. Read more information on sharing protocols at https://plos.org/protocols?utm_medium=editorial-email&utm_source=authorletters&utm_campaign=protocols.

Response 1:

Thank you for this recommendation. The laboratory protocols used in this study, including the formalin-ethyl acetate concentration technique (FECT) for fecal examination and the urine antigen ELISA for detecting Opisthorchis viverrini infection, are well-established methods that have been described in detail and previously published. Appropriate references to these protocols have been provided in the Methods section of the manuscript.

Specifically, the FECT procedure was performed according to the previously published protocol described by Kopolrat et al. (2022), while the urine antigen ELISA was conducted following the methods reported by Worasith et al. (2015) and Worasith et al. (2019).

As the corresponding author, your ORCID iD is verified in the submission system and will appear in the published article. PLOS supports the use of ORCID, and we encourage all coauthors to register for an ORCID iD and use it as well. Please encourage your coauthors to verify their ORCID iD within the submission system before final acceptance, as unverified ORCID iDs will not appear in the published article. Only the individual author can complete the verification step; PLOS staff cannot verify ORCID iDs on behalf of authors.

Response 2:

Thank you for this information. We will encourage all coauthors to register for and verify their ORCID IDs within the submission system prior to final acceptance so that their verified ORCID IDs can be included in the published article.

If the reviewer comments include a recommendation to cite specific previously published works, please review and evaluate these publications to determine whether they are relevant and should be cited. There is no requirement to cite these works unless the editor has indicated otherwise.

Response 3:

Thank you for this guidance. We carefully reviewed all reviewer comments and confirmed that no specific previously published works were recommended for citation. Therefore, no additional references were added in response to this comment.

Please review your reference list to ensure that it is complete and correct. If you have cited papers that have been retracted, please include the rationale for doing so in the manuscript text, or remove these references and replace them with relevant current references. Any changes to the reference list should be mentioned in the rebuttal letter that accompanies your revised manuscript. If you need to cite a retracted article, indicate the article’s retracted status in the References list and also include a citation and full reference for the retraction notice.

Response 4:

Thank you for this guidance. We carefully reviewed all reviewer comments and confirmed that no specific previously published works were recommended for citation. Therefore, no additional references were added in response to this comment.

#Respond to the Editor (highlighted in yellow)

First, please verify the source and license of the underlying geospatial data used in Fig. 1. The response indicates that the figure was created in QGIS, but QGIS is the software used to prepare the map and does not by itself establish that the underlying map layers, boundaries, or other geospatial data are compatible with publication under the CC BY 4.0 license. Please add the actual data source(s) for the map layers to the figure caption and confirm that these data can be redistributed under the journal’s license.

Response 1:

Thank you for this comment. We confirm that QGIS was used solely as the software to prepare Figure 1. The underlying geospatial data consisted of administrative boundary shapefiles obtained from the United Nations Office for the Coordination of Humanitarian Affairs (UN-OCHA) Humanitarian Data Exchange (HDX) geoBoundaries dataset for Thailand. We have revised the figure caption to include the source of the geospatial data and confirmed that these data can be redistributed under the applicable open-data license in accordance with the journal's requirements. (Materials and methods section, lines 108-113)

Second, please harmonize the ethics and consent wording between the manuscript and the submission form. The submission ethics statement indicates that only participants who provided written informed consent were included, whereas the manuscript states that adults provided written informed consent and that parents or legal guardians provided verbal consent for participants aged 15–17 years. Please ensure that the wording is consistent and that the reported form of consent for minors matches the ethics approval.

Response 2:

Thank you for highlighting this inconsistency. We have reviewed the ethics approval documents and revised the manuscript and submission information to ensure consistency in the description of the informed consent procedures. The revised wording now states that written informed consent was obtained from all adult participants and that written informed consent was obtained from the parents or legal guardians of participants aged 15-17 years prior to enrollment in the study. The ethics and consent statements are now fully aligned throughout the manuscript and submission materials. (Materials and methods section, lines 117-120)

Attachment Submitted filename: Response to reviewers_R2.docx

# Editor report: Decision Letter 2

**DOI:** 10.1371/journal.pone.0352854.r005

**Authors:** Petr Heneberg, Petr Heneberg, Petr Heneberg

16 Jun 2026

Longitudinal changes and risk factors of Opisthorchis viverrini infection after selective praziquantel treatment: evidence from urine antigen assay and fecal examination in an endemic community in Northeast Thailand

PONE-D-26-20582R2

Dear Dr. Kopolrat,

We’re pleased to inform you that your manuscript has been judged scientifically suitable for publication and will be formally accepted for publication once it meets all outstanding technical requirements.

Within one week, you’ll receive an e-mail detailing the required amendments. When these have been addressed, you’ll receive a formal acceptance letter and your manuscript will be scheduled for publication.

An invoice will be generated when your article is formally accepted. Please note, if your institution has a publishing partnership with PLOS and your article meets the relevant criteria, all or part of your publication costs will be covered. Please make sure your user information is up-to-date by logging into Editorial Manager at Editorial Manager® and clicking the ‘Update My Information' link at the top of the page. For questions related to billing, please contact billing support.

If your institution or institutions have a press office, please notify them about your upcoming paper to help maximize its impact. If they’ll be preparing press materials, please inform our press team as soon as possible -- no later than 48 hours after receiving the formal acceptance. Your manuscript will remain under strict press embargo until 2 pm Eastern Time on the date of publication. For more information, please contact onepress@plos.org.

Kind regards,

Petr Heneberg

Academic Editor

PLOS One

Additional Editor Comments (optional):

Reviewers' comments:

# Editor report: Acceptance letter

**DOI:** 10.1371/journal.pone.0352854.r006

**Authors:** Petr Heneberg, Petr Heneberg, Petr Heneberg

PONE-D-26-20582R2

PLOS One

Dear Dr. Kopolrat,

I'm pleased to inform you that your manuscript has been deemed suitable for publication in PLOS One. Congratulations! Your manuscript is now being handed over to our production team.

At this stage, our production department will prepare your paper for publication. This includes ensuring the following:

* All references, tables, and figures are properly cited

* All relevant supporting information is included in the manuscript submission,

* There are no issues that prevent the paper from being properly typeset

You will receive further instructions from the production team, including instructions on how to review your proof when it is ready. Please keep in mind that we are working through a large volume of accepted articles, so please give us a few days to review your paper and let you know the next and final steps.

Lastly, if your institution or institutions have a press office, please let them know about your upcoming paper now to help maximize its impact. If they'll be preparing press materials, please inform our press team within the next 48 hours. Your manuscript will remain under strict press embargo until 2 pm Eastern Time on the date of publication. For more information, please contact onepress@plos.org.

You will receive an invoice from PLOS for your publication fee after your manuscript has reached the completed accept phase. If you receive an email requesting payment before acceptance or for any other service, this may be a phishing scheme. Learn how to identify phishing emails and protect your accounts at https://explore.plos.org/phishing.

If we can help with anything else, please email us at customercare@plos.org.

Thank you for submitting your work to PLOS ONE and supporting open access.

Kind regards,

PLOS ONE Editorial Office Staff

on behalf of

Dr. Petr Heneberg

Academic Editor

PLOS One
